# A novel proteinaceous molecule produced by *Lysinibacillus* sp. OF-1 depends on the Ami oligopeptide transporter to kill *Streptococcus pneumoniae*

**DOI:** 10.1099/mic.0.001313

**Published:** 2023-03-07

**Authors:** Ingvild Hals Hauge, Vilde Sandegren, Anja Ruud Winther, Cathrine Arnason Bøe, Zhian Salehian, Leiv Sigve Håvarstein, Morten Kjos, Daniel Straume

**Affiliations:** ^1^​ Faculty of Chemistry, Biotechnology and Food Science, Norwegian University of Life Sciences, 1430 Ås, Norway; ^2^​ Department of Molecular Biology, Norwegian Veterinary Institute, 1433 Ås, Norway

**Keywords:** *Lysinibacillus*, antimicrobials, *Streptococcus pneumoniae*, lysinicin OF

## Abstract

Infections caused by antibiotic-resistant *Streptococcus pneumoniae* are of growing concern for healthcare systems, which need new treatment options. Screening microorganisms in terrestrial environments has proved successful for discovering antibiotics, while production of antimicrobials by marine microorganisms remains underexplored. Here we have screened microorganisms sampled from the Oslo Fjord in Norway for production of molecules that prevent the human pathogen *S. pneumoniae* from growing. A bacterium belonging to the genus *Lysinibacillus* was identified. We show that this bacterium produces a molecule that kills a wide range of streptococcal species. Genome mining in BAGEL4 and AntiSmash suggested that it was a new antimicrobial compound, and we therefore named it lysinicin OF. The compound was resistant to heat (100 °C) and polymyxin acylase but susceptible to proteinase K, showing that it is of proteinaceous nature, but most probably not a lipopeptide. *S. pneumoniae* became resistant to lysinicin OF by obtaining suppressor mutations in the *ami* locus, which encodes the AmiACDEF oligo peptide transporter. We created Δ*amiC* and Δ*amiEF* mutants to show that pneumococci expressing a compromised Ami system were resistant to lysinicin OF. Furthermore, by creating mutants expressing an intact but inactive Ami system (AmiED184A and AmiFD175A) we could conclude that the lysinicin OF activity depended on the active form (ATP-hydrolysing) of the Ami system. Microscopic imaging and fluorescent labelling of DNA showed that *S. pneumoniae* treated with lysinicin OF had an average reduced cell size with condensed DNA nucleoid, while the integrity of the cell membrane remained intact. The characteristics and possible mode of action of lysinicin OF are discussed.

## Introduction

The growing numbers of antibiotic-resistant pathogens are a global concern, threatening several aspects of modern medicine [1, 2]. Efficient antibiotics are critical not only to treat bacterial infections in general, but also to prevent infections after surgeries and in patients undergoing chemotherapy. Efforts to slow the spread of antibiotic resistance among pathogens include restrictive use of antibiotics by the medical healthcare systems as well as in the agricultural industry. Combined with vaccination programmes and the use of narrow-range antibiotics, the spread of antibiotic resistance can be slowed. However, these measures do not offer a final solution to the problem. Therefore, to have treatment options for bacterial infections in the future, there is a need to discover new antimicrobial compounds with the potential for clinical use. *Streptococcus pneumoniae *, also called pneumococcus, is a human pathogen causing pneumonia, bacteraemia, meningitis and otitis media [3]. Children, the elderly and immunocompromised individuals are particularly susceptible to being infected with pneumococci. Penicillin is the antibiotic of choice, but its clinical relevance is fading due to increasing numbers of infections caused by penicillin-resistant strains. In addition, several pneumococcal isolates are reported to be resistant to macrolides, fluoroquinolones and tetracyclines [4]. Since *S. pneumoniae* can become competent for natural genetic transformation, the resistance genes can be rapidly spread to susceptible strains, adding an extra layer to this challenge [5].

In this work we set out to find new natural compounds that inhibited the growth of *S. pneumoniae *. A large number of antibiotics used today, e.g. β-lactams, macrolides and tetracyclines, are natural products produced by other microorganisms found in terrestrial habitats [6, 7]. Production of antimicrobials by marine microorganisms, however, is underexplored. Therefore, we sampled microorganisms from the shore of the Oslo Fjord in Norway and screened them for production of anti-pneumococcal activity. Here we describe an isolate belonging to the genus *Lysinibacillus* that produces a novel compound inhibiting *S. pneumoniae* and other streptococcal species. We showed that this compound, which we named lysinicin OF, depends on the Ami oligopeptide uptake system to kill *S. pneumoniae* by an unknown mechanism. The biophysical properties and mode of action of lysinicin OF are discussed.

## Methods

### Bacterial strains, growth conditions and transformation

All bacterial species and mutants used in this study are listed in Table S1 (available in the online version of this article). *S. pneumoniae* was grown in liquid C medium [8] without shaking and on Todd Hewitt (TH) agar (Becton, Dickinson and Company) at 37 °C. Other streptococcal species were grown in TH broth and on TH agar. When grown on TH agar, all streptococci were incubated anaerobically by placing them in an airtight container containing AnaeroGen bags from Oxoid. Growth curves of pneumococcal strains were obtained by growing them in 96-well microtitre plates using a Hidex Sense Microplate Reader. All strains were pregrown for 1 h in C medium before they were diluted to OD_550_=0.05 in fresh C medium and transferred to a microtitre plate. In some cases, a final concentration of 2 µm Sytox Green (Thermo Fisher Scientific) was added to the wells for detection of cell lysis. Sytox Green fluoresces upon DNA binding, which it only obtains access to if the cell membrane integrity is disrupted. Sytox Green was excited at 485 nm and the light emission at 535 nm was measured. *Escherichia coli* was grown in lysogeny broth (LB) with shaking. *Bacillus subtilis *, *Staphylococcus aureus *, *Pseudomonas brenneri* and *Mycobacterium smegmatis* were grown in brain heart infusion (BHI) broth (Oxoid). *B. subtilis *, *S. aureus* and *M. smegmatis* were incubated at 37 °C with shaking, while *P. brenneri* was incubated at 22 °C without shaking. *Lactococcus lactis* was grown in GM17 (Oxoid) at 30 °C without shaking, and *Enterococcus faecalis* was grown in BHI broth at 37 °C without shaking. *Lysinibacillus* sp. OF-1 was grown aerobically in TH broth, in M9 medium [supplemented with a final concentration of 0.4 % (v/v) glucose, 10 µg ml^−1^ (w/v) of all 20 amino acids, 1 mM MgSO_4_, 0.1 mM CaCl_2_ and 1 µg ml^−1^ (w/v) thiamine] and on TH agar at room temperature. Liquid cultures of *Lysinibacillus* sp. OF-1 were grown without shaking, but a maximum liquid depth of 3 cm allowed sufficient aerobic conditions.

*S. pneumoniae* was transformed by mixing 1 ml of exponentially growing cells (OD_550_ between 0.05 and 0.1) with 100–200 ng of transforming DNA and CSP-1 (final concentration of 250 ng ml^−1^). The transforming cells were incubated at 37 °C for 2 h before 30 µl of the cell culture was plated on TH agar containing a final concentration of 400 µg ml^−1^ kanamycin, 200 µg ml^−1^ streptomycin, 200 µg ml^−1^ spectinomycin or 2.5 µg ml^−1^ chloramphenicol.

### Sampling and soft agar overlay assay

Samples were collected from the Oslo Fjord at the shore near a small village called Hvitsten. Samples from rocks, seaweed, sand, mud and seawater were spread onto BHI, LB, TH and Mueller–Hinton agar (Becton, Dickinson and Company) and incubated at room temperature for 5 days to obtain bacterial colonies. A volume of 100 µl *S *. *pneumoniae* culture with OD_550_=0.3 was added to 5 ml of melted TH soft agar [0.75 % (w/v) agar] holding 47 °C. The soft agar was then mixed by vortexing for 2 s, before it was gently spread on top of colonies formed by the marine bacteria as described above. After anaerobic incubation at 37 °C overnight, the plates were inspected for colonies surrounded by inhibition zones. For detection of inhibition zones surrounding *Lysinibacillus* sp. OF-1 colonies, the same protocol with TH soft agar was used for all streptococcal indicator species. BHI soft agar was used when *P. brenneri *, *B. subtilis *, *S. aureus* and *M. smegmatis* were the indicators, while LB soft agar was used for *E. coli* and GM17 soft agar for *L. lactis* and *E. faecalis *.

### DNA techniques

All primers used for PCR are listed in Table S2. Gene cassettes used for transformation of *S. pneumoniae* were created by overlap extension PCR [9]. To make gene deletion cassettes, the ~1000 bp regions upstream and downstream of a gene of interest were fused to the 5′ and 3′ end of a desired antibiotic resistance gene (Kan^r^, Spc^r^, Cam^r^). A Janus cassette [10] was used to introduce gene deletions or mutations. When appropriate, the Janus was replaced through negative selection with a DNA sequence of interest by fusing it with the same ~1000 bp regions flanking the Janus. Point mutations and fusion tags were introduced by primer design and overlap extension PCR.

### Lysinicin OF enrichment

*Lysinibacillus* sp. OF-1 was cultivated for 4 days in 500 ml TH broth. Cells were removed by centrifugation at 5000 ***g***, and the supernatant was transferred to an Erlenmeyer flask containing 3 g of Amberlite XAD16N 20–60 mesh beads (Sigma). The flask was incubated at room temperature with shaking for 1 h. The beads were then washed twice with water and once with 20 % ethanol before the remaining material bound to the beads was eluted by 3×5 ml 96 % ethanol. The three elution fractions were pooled and dried by vacuum centrifugation. The dried material was dissolved in 1 ml sterile water and stored at −20 °C.

### Whole-genome sequencing and genome analyses

Genomic DNA from bacteria was isolated by using NucleoBond AXG100 columns as described in the included protocol from Macherey-Nagel. Resequencing of gDNA from pneumococcal mutants was performed by using MiSeq nano v2 with paired ends reads of 250 bp yielding approximately 35× coverage. Genomic DNA from *Lysinibacillus* sp. OF-1 was subjected to both short- (Illumina) and long-read (Nanopore) sequencing. Illumina sequencing of *Lysinibacillus* sp. OF-1 was performed using v3 chemistry on the MiSeq with paired end reads of 300 bp yielding 80× coverage. For long-read sequencing a library was prepared from 400 ng of gDNA using the Rapid Barcoding kit SQK-RBK004 from Oxford Nanopore Technologies. This library was further sequenced on the MinION sequencer using the FLO-Min106D flowcell (Oxford Nanopore Technologies). Fast5 files generated from nanopore sequencing were used for basecalling with Guppy (version 4.0.15). Quality control of the sequencing run was performed with NanoPlot (version 1.33.1). Fastq files from nanopore sequencing were demultiplexed with Qcat (version 1.1.0), where barcodes were trimmed and reads shorter than 50 bp were excluded. The demultiplexed fastq files were then processed with NanoFilt (version 2.7.1) to remove reads with a Phred score quality <7 and a minimum length <100 bp. In addition, the first 50 bp of all reads were removed using the headcrop option. *De novo* assembly of the *Lysinibacillus* sp. OF-1 genome was performed via hybrid assembly of the Illumina and Nanopore sequences using the Ellipsis pipeline (10.5281/zenodo.4563897). Annotation of the *Lysinibacillus* sp. OF-1 genome was performed using prokka 1.14.5 [11].

### Microscopic analyses

For microscopic imaging, bacteria were immobilized on a thin layer (<0.5 mm) of 1.2 % (w/v) agarose in phosphate-buffered saline (PBS). Phase contrast and fluorescence pictures were taken using a Zeiss AxioObserver with ZEN Blue software, an ORCA-Flash 4.0 v2 Digital CMOS camera (Hamamatsu Photonics) and a 100× phase-contrast objective. An HXP 120 Illuminator (Zeiss) served as light source for fluorescence microscopy. To stain bacterial nucleoids, cells were treated with a final concentration of 0.2 µg ml^−1^ 4′,6-diamidino-2-phenylindole (DAPI) for 5 min prior to imaging. For live/dead staining (Live/Dead *Bac*Light, Thermo Fisher Scientific) 3 µl of a 1 : 1 mixture of propidium iodide (20 mM) and Syto 9 (3.34 mM) were added to 1 ml cell culture followed by incubation for 15 min in the dark before imaging. Images were analysed using ImageJ software with the MicrobeJ plugin [12].

### Immunoblotting

Pneumococcal mutants were grown to OD_550_=0.25 in 10 ml volumes. Cells were harvested at 4000 ***g*** and lysed in 200 µl SDS sample buffer at 95 °C for 5 min. Total protein extracts from 15 µl samples were separated by SDS-PAGE using a 12 % separation gel and the protocol of Laemmli [13]. Proteins were transferred onto a PVDF membrane using the Trans-Blot Turbo Transfer System from BioRad. FLAG-tagged proteins were detected as described by Stamsås *et al*. [14] using the polyclonal anti-FLAG antibody (Sigma, cat. F7425) and horseradish peroxidase-conjugated anti-rabbit (Thermo Fisher, cat. 31460) as secondary antibody. Both antibodies were diluted 1 : 4000 in TBS-T.

### Haemolysis assay

Sheep blood (Thermo Fisher) was first diluted 1 : 9 in PBS before erythrocytes were collected at 1500 ***g*** for 10 min. The erythrocytes were then washed twice in PBS and finally resuspended in a volume of PBS, resulting in a 1 : 9 dilution of the blood. Aliquots of 990 µl diluted blood were transferred to Eppendorf tubes containing 10 µl lysinicin OF (final concentration of 10× MIC), 10 µl Triton X-100 [final concentration of 1 % (v/v)] or 10 µl PBS. The blood samples were incubated at 37 °C for 30 min. Next, intact erythrocytes were removed from the solution at 1500 ***g*** for 10 min and Abs490 of the supernatants were measured.

### Spot assay

Exponentially growing *S. pneumoniae* at OD_550_=0.2 were treated with 10× MIC lysinicin OF (10 µl ml^−1^), rifampicin (0.39 µg ml^−1^), ciprofloxacin (12.5 µg ml^−1^), ampicillin (3.1 µg ml^−1^) or tetracycline (3.1 µg ml^−1^) for 30 min before the cells were washed three times in fresh C medium. Next, the cells were resuspended in C medium to OD_550_=0.1 and diluted in a 10-fold dilution series of C medium. Three microlitres of the dilutions 10^−1^–10^−6^ were spotted on TH agar. When the spotted dilutions had dried, the plate was incubated at 37 °C for 18 h.

## Results

### A *Lysinibacillus* isolate showed antimicrobial activity against *S. pneumoniae*

**Fig. 1.**
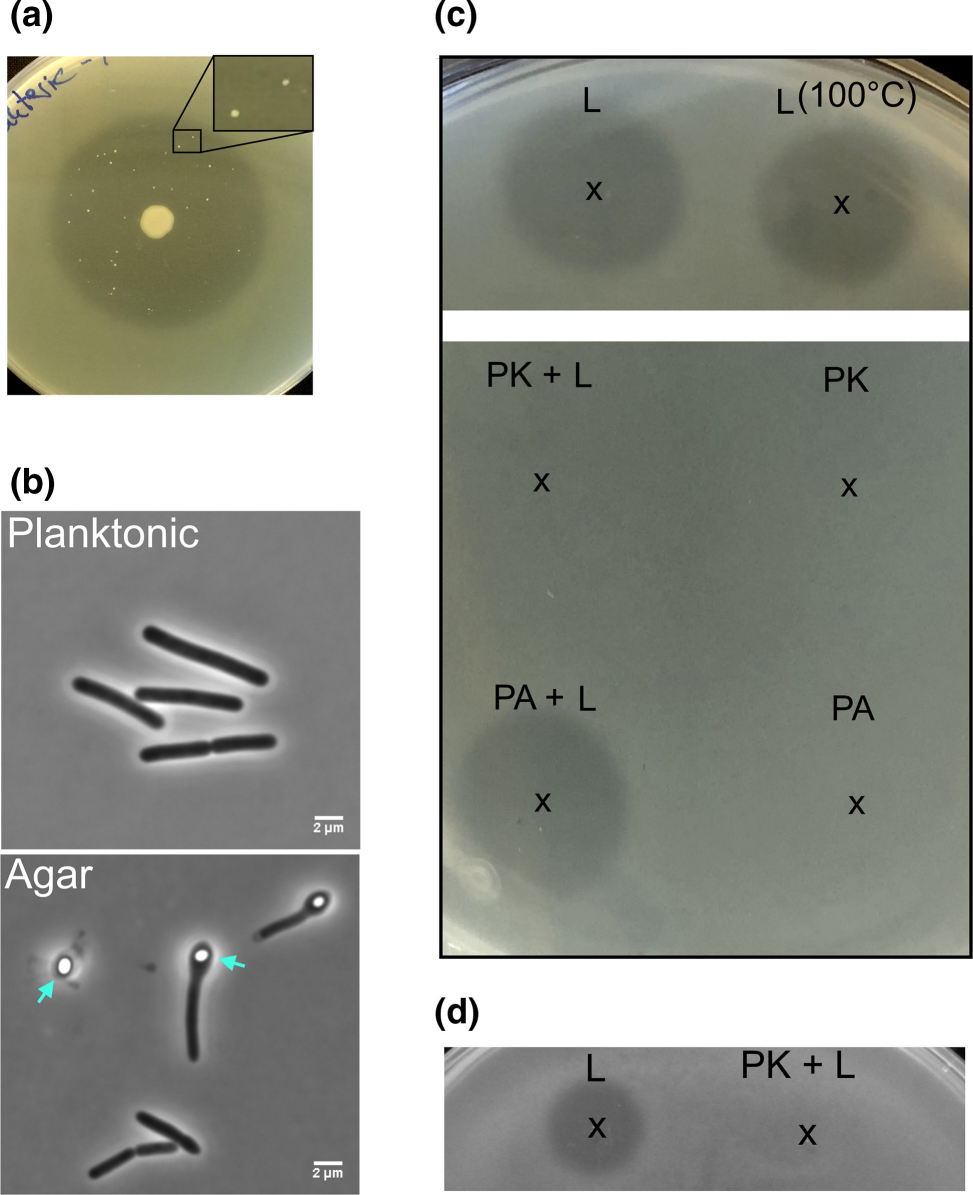
*
Lysinibacillus* sp. OF-1 produces a proteinaceous compound that inhibits *S. pneumoniae *. (a) A colony of *Lysinibacillus* sp. OF-1 overlaid with TH soft agar containing *S. pneumoniae *. Growth inhibition of *S. pneumoniae* is seen as a clear zone surrounding the *Lysinibacillus* sp. OF-1 colony. Several resistant pneumococcal mutants emerged in the inhibition zone after prolonged (48 h) incubation at 37°C (zoomed panel). (b) Phase contrast images of *Lysinibacillus* sp. OF-1 grown in liquid culture (planktonic) and overnight on TH agar. Endospores are indicated by arrows. Scale bars, 2 µm. (c) *S. pneumoniae *-containing soft agar on which 3 µl of lysinicin OF (L) exposed to different treatments was spotted (indicated by x). Lysinicin OF was either incubated at 100°C for 30 min, incubated with 500 µg ml^−1^ proteinase K (PK+L) or 50 µg ml^−1^ polymyxin acylase (PA+L). Proteinase K and polymyxin acylase (PK and PA) had no inhibitory effect on their own. (d) Activity of nontreated and proteinase K-treated lysinicin OF extracted from culture supernatants of *Lysinibacillus* sp. OF-1 grown in minimal M9 medium.

We wanted to explore whether bacteria from the marine habitat have the potential to produce novel antimicrobial compounds against *S. pneumoniae *. Bacteria were sampled from the shore in the Oslo Fjord, including rocks, sand, mud, seaweed and seawater. The samples were plated on TH, BHI, LB and Mueller–Hinton media and incubated aerobically at room temperature for 5 days. To screen for antimicrobial activity against *S. pneumoniae *, the RH425 strain (R6 derivate) was included in TH soft agar placed on top of the marine bacteria. After anaerobic incubation overnight, RH425 inhibition zones were identified. One bacterial colony sampled from the surface of a rock showed a particularly large inhibition zone on TH agar (Fig. S1). A pure culture of this bacterium was obtained and a new soft agar overlay assay was performed to verify the inhibition of *S. pneumoniae* (Fig. 1a).

This marine bacterium did indeed display strong inhibition of *S. pneumoniae *. To identify the isolate, we sequenced its genome and used the 16S rRNA sequence in a blastn search. The best hits were *Lysinibacillus sphaericus* KCCM 35418 and *Lysinibacillus fusiformis* NEB 1292 (100 and 99.87 % identity, respectively), both belonging to the family *Bacillaceae *. Pair-wise genome sequence alignments using blast2 showed that the KCCM 35418 genome covered 77 % of the marine isolate’s genome with a nucleotide identity of 93.09%, while the NEB 1292 genome showed 80 % coverage with 95.36 % nucleotide identity. This shows that the isolate is closely related to *L. sphaericus* and *L. fusiformis *, but also that a large portion of its genetic content is not found in these species.

The genus *Lysinibacillus* currently holds 23 species [National Center for Biotechnology Information (NCBI) taxonomy ID 400634]. To place the isolate in this genus, a phylogenetic tree was generated (Fig. S2) using the M1CR0B1AL1Z3R tool [15] and a selection of 31 genome sequences from NCBI GenBank, including all 23 *Lysinibacillus* species. The phylogram placed the *Lysinibacillus* isolate on the same clade as *L. fusiformis* NEB 1292, confirming a close evolutionary relationship between these two bacteria. However, based on the 16S rRNA and genome sequence analyses (77–80 % coverage), it was difficult to distinguish whether the isolate was an *L. fusiformis* or an *L. sphaericus *, and we have therefore named it *Lysinibacillus* sp. strain OF-1 (for Oslo Fjord isolate 1). It has a genome size of 4 710 095 nucleotides with a GC content of 37.8 % (accession number in GenBank: CP102798). Annotation using prokka 1.14.5 [11] predicted 4658 genes. *Lysinibacillus* sp. OF-1 is a typical rod-shaped bacillus that frequently formed endospores when grown on TH agar (Fig. 1b).

### *Lysinibacillus* sp. OF-1 produces a proteinaceous compound that kills *S. pneumoniae* and other streptococci

To identify *Lysinibacillus* sp. OF-1 gene clusters potentially involved in the production of already known antimicrobial compounds, genome mining was performed using BAGEL4 [16] and AntiSMASH 6.1.1 [17]. BAGEL4 is used to identify biosynthetic gene clusters involved in the production of RiPPs (ribosomally synthesized and posttranslationally modified peptides), while AntiSMASH in addition finds gene clusters involved in synthesis of other secondary metabolites with known antimicrobial activity. No clear hits were found with BAGEL4, whereas AntiSMASH gave a similarity hit of 46 % (percentage of genes found in known gene clusters) to a gene cluster in *Bacillus velezensis* FZB42 responsible for synthesis of the cyclic lipopeptide fengycin (Fig. S3). However, the *Lysinibacillus* sp. OF-1 genome lacks the *fenABCDE* genes (fengycin synthetase A–E) [18], strongly suggesting that the bacterium does not produce a fengycin-like molecule.

To examine the physico-biochemical properties of the antimicrobial compound produced by *Lysinibacillus* sp. OF-1, we first tried to concentrate it from culture supernatants by using hydrophobic XAD Amberlite 16 N beads (see Methods). Indeed, the material eluted from the XAD beads inhibited *S. pneumoniae* (Fig. 1c), demonstrating hydrophobic properties of the compound. Since species belonging to the genus *Bacillus* often produce antimicrobial proteins, peptides and lipopetides [19, 20], we tested how proteinase K, polymyxin acylase and heat treatment would affect the antimicrobial activity of the compound. Neither heat treatment at 100 °C for 30 min nor incubation with polymyxin acylase (cleaving the acyl bond between the peptide and lipid part of lipopeptides) reduced its antimicrobial effect on *S. pneumoniae *. Treatment with proteinase K, on the other hand, completely inactivated the antimicrobial activity (Fig. 1c). This demonstrated that the antimicrobial compound is of proteinaceous nature but most likely not a lipopeptide. Considering that it retained inhibitory activity after 100 °C for 30 min, we reasoned that the compound could be a peptide or possibly a glycopeptide. The latter seemed less likely since antimicrobial glycopeptides (e.g. vancomycin, teicoplanin and balhimycin) are usually produced by bacteria belonging to Actinomycetia [21] and that we did not find glycopeptide gene clusters in the *Lysinibacillus* sp. OF- 1 genome. We named the compound lysinicin OF. To confirm that lysinicin OF was produced by *Lysinibacillus* sp. OF-1 and not a degradation product derived from consumption of the TH-medium, we also successfully enriched the compound from the supernatant of *Lysinibacillus* sp. OF-1 grown in M9 mineral medium supplemented with amino acids (see Methods) (Fig. 1d). Using UHPLC and matrix-assisted laser desorption/ionization time-of-flight tandem mass spectrometry (MALDI TOF MS-MS), we have made several attempts to identify the mass and amino acid composition of the lysinicin OF extracted from both TH and M9 medium, however, thus far, none have been successful. For HPLC-fractionated TH supernatants, we found only peptides deriving from the growth medium, while in M9 supernatants we obtained many peaks of different masses. We were not able to pinpoint which of the masses that represented the active compound.

To determine whether lysinicin OF had a target range beyond the pneumococcus, we performed soft agar overlay assays using a selection of streptococci covering species from all six streptococcal subgroups (Mitis, Pyogenes, Anginosus, Mutans, Bovis and Salivarius) as well as more distantly related species such as *M. smegmatis *, *B. subtilis *, *S. aureus *, *L. lactis *, *E. faecalis *, *P. brenneri* and *E. coli* as indicators. The results are presented in Table 1 (see Fig. S4a for overlay assays). All streptococci tested displayed various degrees of sensitivity to lysinicin OF, except for *Streptococcus agalactiae* NCTC8181 from the pyogenes group, which had no inhibition zone. However, another representative from the pyogenes group, *Streptococcus phocae* ATCC29128, was sensitive, showing that species within all six subgroups of streptococci were sensitive to lysinicin OF. In addition, lysinicin OF displayed weak inhibitory effect against *B. subtilis *, but not against *M. smegmatis *, *S. aureus *, *L. lactis *, *E. faecalis *, *P. brenneri* and *E. coli *.

**Table 1.** Bacteria tested for lysinicin OF sensitivity in soft agar overlay assays

Species	Sensitive	Streptococcal group	Source
* S. pneumoniae* R6	Yes	Mitis	J.P. Claverys
* S. pneumoniae* D39	Yes	Mitis	[70]
* S. mitis* SK142	Yes	Mitis	M. Kilian
* S. oralis* ATCC10557	Yes	Mitis	M. Kilian
* S. peroris* SK958	Yes	Mitis	M. Kilian
* S. infantis* SK140	Yes	Mitis	M. Kilian
* S. sanguinis* SK90	Yes	Mitis	M. Kilian
* S. parasanguinis* ATCC15912	Yes	Mitis	M. Kilian
* S. gordonii* SK6	Yes	Mitis	M. Kilian
* S. cristatus* NCTC12479	Yes	Mitis	M. Kilian
* S. vestibularis* NCTC 12166	Yes	Salivarius	M. Kilian
* S. bovis* NCTC8177	Yes	Bovis	M. Kilian
* S. agalactiae* NCTC8181	No	Pyogenes	M. Kilian
* S. phocae* ATCC29128	Yes	Pyogenes	M. Kilian
* S. criceti* ATCC19642	Yes	Mutans	M. Kilian
* S. mutans* NCTC10449	Yes	Mutans	M. Kilian
* S. anginosus* SK87	Yes	Anginosus	M. Kilian
* B. subtilis* ATCC6051	Moderate	na	ATCC
* M. smegmatis* NCTC8159	No	na	UKHSA
* E. coli* DH5a	No	na	Invitrogen
* P. brenneri *	No	na	Lab stock, This study
* L. lactis* MG1363	No	na	Lab stock
* S. aureus* NCTC8325	No	na	Lab stock
* E. faecalis* LMG2708	No	na	Lab stock

NA - Not Applicable

### Inactivation of the Ami oligopeptide transporter renders *S. pneumoniae* immune against lysinicin OF

**Fig. 2.**
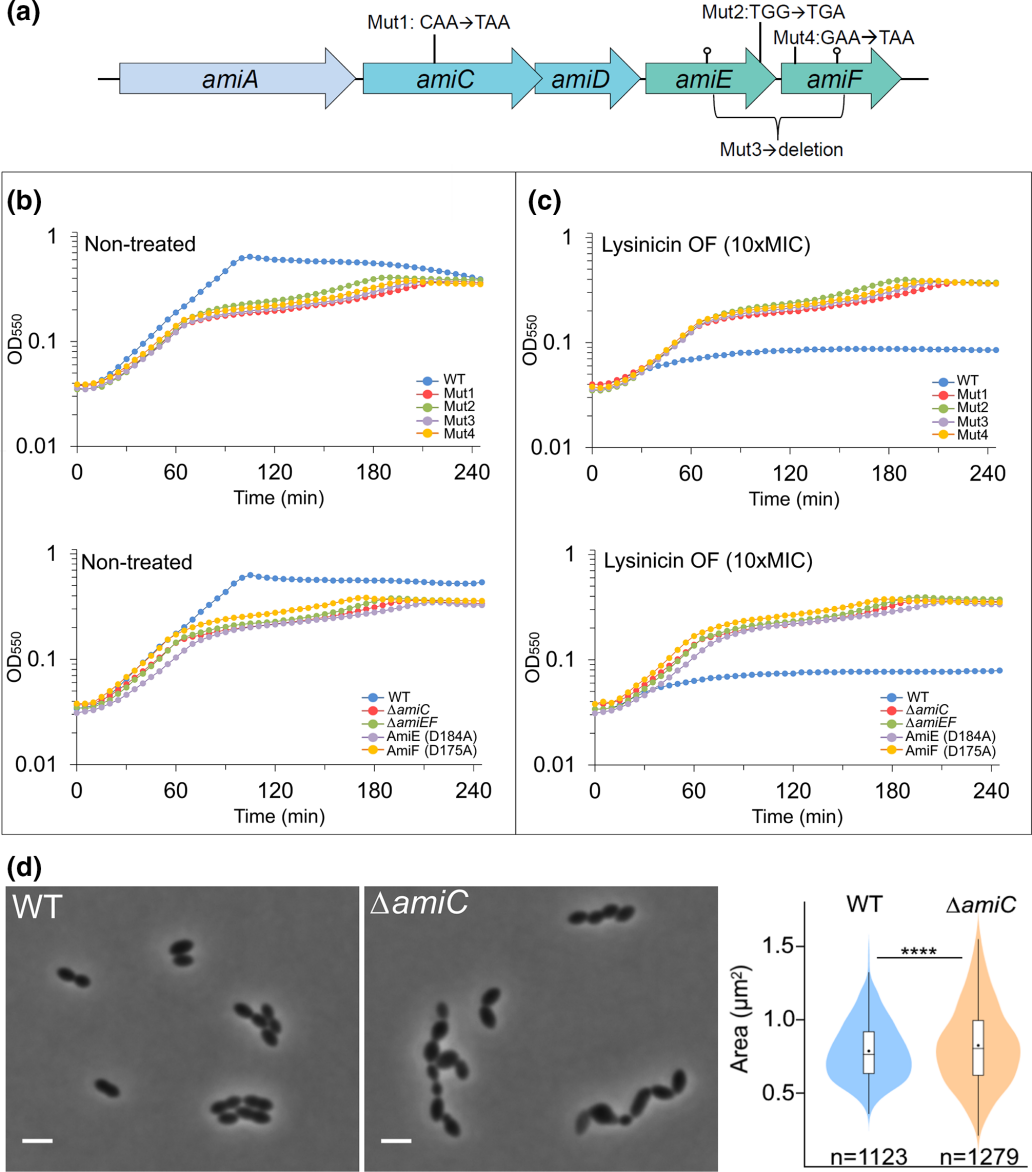
Lysinicin OF sensitivity of *S. pneumoniae ami* mutants. (**a**) Schematic diagram of the *ami* operon depicting the mutations in mutants 1–4 (see Table 2) and the codon positions of D184 and D175 in *amiE* and *amiF* (lollipops). Mutants 1–4, Δ*amiC*, Δ*amiEF*, *amiE^D184A^* and *amiF^D175A^*, and wild-type cells (strain RH425) were grown without (**b**) or in the presence (**c**) of 10 µl ml^−1^ lysinicin OF (10× MIC). All mutants expressing a compromised Ami system displayed resistance towards lysinicin OF, whereas the growth of the wild-type was significantly inhibited. (**d**) The lysinicin OF-resistant Δ*amiC* mutant displayed cells of heterogenous sizes with an average size of 0.83±0.27 µm^2^ compared with 0.79±0.20 µm^2^ for wild-type. *P*-values were obtained relative to wild-type using one-way analysis of variance (ANOVA) analysis, ****P<0.0001. Scale bars, 2 µm.

Our results suggested that lysinicin OF could be an antimicrobial peptide with similar characteristics to bacteriocins. Bacteriocins of Gram-positive bacteria typically recognize specific receptor molecules on the surface of their target bacteria. Once bound to the receptor, the bacteriocins form a lethal pore in the cell membrane, either by themselves or in complex with the receptor [22–29]. To identify a potential receptor of lysinicin OF, we checked whether *S. pneumoniae* could develop resistance to the compound. As described above, a soft agar overlay containing *S. pneumoniae* resulted in a large inhibition zone surrounding the spotted colony of *Lysinibacillus* sp. OF-1 (Fig. 1a). The plate was incubated at 37 °C for a prolonged period and after 48 h we observed several colonies within the inhibition zone (Fig. 1a). Four of them were picked and restreaked on TH agar to make pure cultures. After identifying them as *S. pneumoniae* by 16S rRNA sequencing, their tolerance to lysinicin OF was tested in both soft agar overlays and in liquid cultures (Figs 2 and S4b). In these experiments all four isolates showed full immunity against lysinicin OF, even when grown with a concentration 10 times higher than the relative MIC determined for lysinicin OF (Fig. S5). The resistant mutants were named mutants 1, 2, 3 and 4. The lysinicin OF resistance, however, appeared to come with a fitness cost, since all four mutants displayed reduced growth compared to the parental wild-type strain (Fig. 2). To identify these mutations, we sequenced the whole genome of mutants 1–4 and mapped the reads to the R6 reference genome (NC_003098.1). Strikingly, all had mutations in the *ami* operon (Table 2 and Fig. 2a). The *amiACDEF* genes code for an oligopeptide uptake system of the ATP-binding cassette (ABC) transporter type [30, 31]. In *S. pneumoniae* this system has been shown to transport peptides of 2–7 amino acids, although longer peptides have not been tested for this species [32]. The Ami system in *S. thermophilus *, on the other hand, has been shown to internalize peptides of up to 23 amino acids [33].

**Table 2.** Mutations found in lysinicin OF-resistant *S. pneumoniae* mutants

Mutant no.	Mutation type	Position on ref. genome (NC_003098.1)	Affected gene(s)	Consequences
1	SNP, C→T	1 676 261	*amiC*	Stop codon
2	SNP, G→A	1 673 543	*amiE*	Stop codon
3	Deletion	Δ1672934–1 674 011	*amiEF*	Disrupted AmiE and AmiF
4	SNP, G→T	1 673 313	*amiF*	Stop codon

In the Ami system, the lipoprotein AmiA binds extracellular peptides and passes them on to a peptide translocation channel composed of the two non-homologous membrane proteins, AmiC and AmiD. Peptides bound to AmiCD are then internalized, most probably because the AmiCD channel undergoes a conformational change powered by ATP hydrolysis by the two associated cytoplasmic ATPases, AmiE and AmiF [30]. Mutants 1, 2 and 4 had a point mutation in *amiC*, *amiE* and *amiF*, respectively, that resulted in premature termination of mRNA translation (stop codon in codons 223, 318 and 36, respectively). Mutant 3 had a deletion that removed the end of *amiE* and the beginning of *amiF* (the DNA sequence coding for the last 147 amino acids of AmiE and the first 161 amino acids of amiF was deleted). All four mutations thus most probably resulted in an inactive Ami system, which could no longer import extracellular peptides.

To confirm that inactivation of the Ami system results in resistance to lysinicin OF, we used the sensitive RH425 strain and created Δ*amiC* and a Δ*amiEF* mutants and treated them with lysinicin OF. Similar to mutants 1–4, inactivation of the Ami system (Δ*amiC* and Δ*amiEF*) resulted in full immunity against lysinicin OF and reduced growth (Figs 2 and S4b). Reduced growth of Ami mutants has also been previously reported by Alloing and co-workers [30]. Microscopic examination of the Δ*amiC* mutant revealed severe morphological abnormalities (Fig. 2d), corroborating the fitness cost that comes with lysinicin OF resistance.

### Bactericidal effect of lysinicin OF depends on ATP hydrolysis by the Ami system

Resistance to lysinicin OF was obtained by disruption of the Ami system. This suggests that the Ami system functions as a lysinicin OF receptor to facilitate pore formation in the cell membrane, or alternatively, that lysinicin OF is taken up via the Ami system to execute its lethal action inside the cell. Translocation of extracellular peptides across the membrane depends on the ATPases AmiE and AmiF, which consume ATP to induce a conformational change in the AmiCD permease [30, 34]. To test which of the abovementioned hypotheses is true, we created two mutants in which the Ami systems were kept intact except for their ability to hydrolyse ATP. We reasoned that if such mutants were still sensitive, lysinicin OF most likely uses the Ami system as a membrane-embedded receptor. On the other hand, if these mutants became resistant to lysinicin OF, it supports the idea that lysinicin OF must enter the cell/cytoplasm to become lethal. Both AmiE and AmiF have the typical Walker A (GxxxxGKS/T, where x can be various amino acids) and Walker B [hhhhD(D/E), where h is hydrophobic amino acids] motifs found in ATPases (Fig. S6). Walker A primarily binds ATP, while Walker B executes ATP hydrolysis [35–37]. The Walker B motif contains a conserved aspartic acid residue, which coordinates a Mg^2+^ ion essential for ATP hydrolysis [37]. Substitution of this aspartic acid residue with alanine in homologous ATPases has been shown to inactivate their ATPase activity [38, 39]. By aligning the amino acid sequences of AmiE and AmiF against a homologous ATPase (DppD) from *Caldanaerobacter subterraneus* sp. *tengcongensis *, for which the 3D structure in complex with ATP and Mg^2+^ has been solved (Fig. S6), Asp184 was identified in AmiE and Asp175 in AmiF. We created two mutants, one in which the native AmiE was replaced with AmiE(D184A) and another in which AmiF was replaced with AmiF(D175A). Similar to all the other lysinicin OF-resistant mutants, they grew more slowly than wild-type cells, in addition to being resistant to lysinicin OF (Figs 2 and S4). This showed that an intact but inactive Ami system makes *S. pneumoniae* resistant to lysinicin OF, supporting that the compound is taken up by the cells. It is worth noting that this phenotype could not be attributed to destabilization of the mutated AmiEF proteins, since immunoblotting showed that the expression levels of the mutated variants were comparable with wild-type AmiEFs (Fig. S7).

To further understand how the Ami system affects lysinicin OF activity, we exposed a Δ*amiA* mutant to lysinicin OF (AmiA binds extracellular peptides and provides them to the AmiCDEF complex for import) [30, 32]. Unexpectedly, AmiA-deficient cells displayed similar sensitivity to lysinicin OF as the wild-type cell (Fig. S8). In contrast to our previous result, this indicates that lysinicin OF is not imported into the cytoplasm to kill the target cells, although an ATP-consuming Ami system is a prerequisite for its activity. To exclude the possibility of redundancy by the AmiA paralogues AliA and AliB [32], we tested the lysinicin OF susceptibility of a Δ*amiA*, Δ*aliA*, Δ*aliB* triple mutant. Similar to the wild-type, this mutant was also sensitive (Fig. S8). Together, this shows that the activity of lysinicin OF depends on an intact AmiCD permease and active ATP hydrolysis by AmiEF; however, the peptide-binding protein AmiA is not involved in the mechanism of action.

### Lysinicin OF does not disturb the cell membrane integrity

**Fig. 3.**
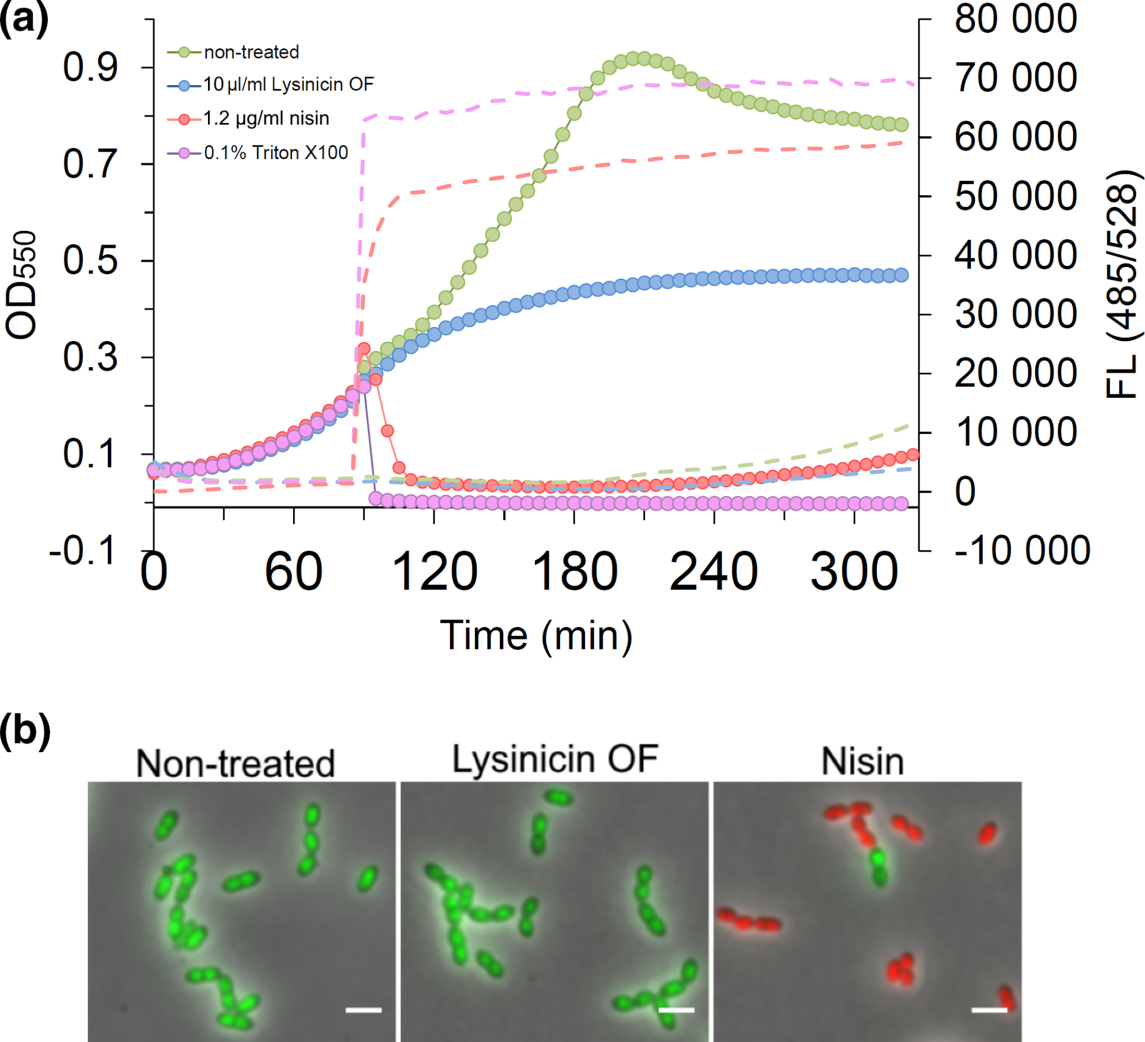
The effect of lysinicin OF on cell membrane integrity. (**a**) *S. pneumoniae* was grown in the presence of the nucleic acid stain Sytox Green, which fluoresces upon DNA binding when excited at 485 nm. Since Sytox Green is unable to cross intact cell membranes, an increase in fluorescent signal is directly correlated with reduced cell membrane integrity. At OD_550_=0.25 lysinicin OF was added to a final concentration of 10× MIC. The optical density (circles) and fluorescent signal (dotted lines) were recorded every fifth minute. The pore-forming peptide nisin (10× MIC) and the detergent Triton X-100 (0.1%, v/v) were used as known membrane interfering controls. (**b**) Dead/live staining of *S. pneumoniae* (Δ*lytA*) treated with 10× MIC lysinicin OF or nisin for 30 min. Cells with intact cytoplasmic membranes appear green, while cells with a leaky cell membrane are red. Scale bars, 2 µm.

One of our hypotheses was that lysinicin OF uses the AmiCDEF complex as a docking molecule in order to interfere with the cytoplasmic membrane of target cells. We did observe that the toxic effect of lysinicin OF was nearly irreversible, i.e. inhibiting cell growth without the possibility of recovering after lysinicin OF removal (Fig. S9). This is compatible with a model where lysinicin OF induces a biophysical change in the cells, e.g. interfering with the membrane integrity. To further test this hypothesis, we determined whether the membrane of lysinicin OF-treated cells became permeable to the fluorescent dye Sytox Green. This fluorophore fluoresces upon binding DNA, but it is not able to cross intact cell membranes. Hence, disintegration of the cell membrane can be detected as increased fluorescence. *S. pneumoniae* RH425 were grown in the presence of 2 µm Sytox Green. At OD_550_=0.25, lysinicin OF was added to a final concentration of 10× the relative MIC value. Nisin was used as a control (see Fig. S5 for MIC values) representing a pore-forming peptide [40–42] and Triton X-100 as a membrane-dissolving detergent. After addition of lysinicin OF, the cell growth levelled out within an hour, but no increase in fluorescence was detected (Fig. 3a). Nisin and Triton X-100, on the other hand, resulted in reduced cell densities and a significant increase in fluorescence, showing that pneumococcal autolysis is also induced when the membrane integrity is disrupted. Similarly, dead/live staining and fluorescence microscopy showed that the cytoplasmic membrane is intact after exposure to 10× MIC of lysinicin OF for 30 min (Fig. 3b). The pore-forming peptide nisin, on the other hand, clearly permeabilized the cytoplasmic membrane. Since no membrane-destabilizing effect was detected for *S. pneumoniae* upon lysinicin OF treatment, we checked whether eukaryotic cell membranes could also tolerate this compound. A common obstacle of many new antimicrobials is that they disrupt eukaryotic cell membranes, causing haemolysis [43, 44]. In the case of lysinicin OF, however, treatment of sheep blood cells with 10× MIC of lysinicin OF did not lead to haemolysis (Fig. S10).

### Morphology of lysinicin OF-treated pneumococci

**Fig. 4.**
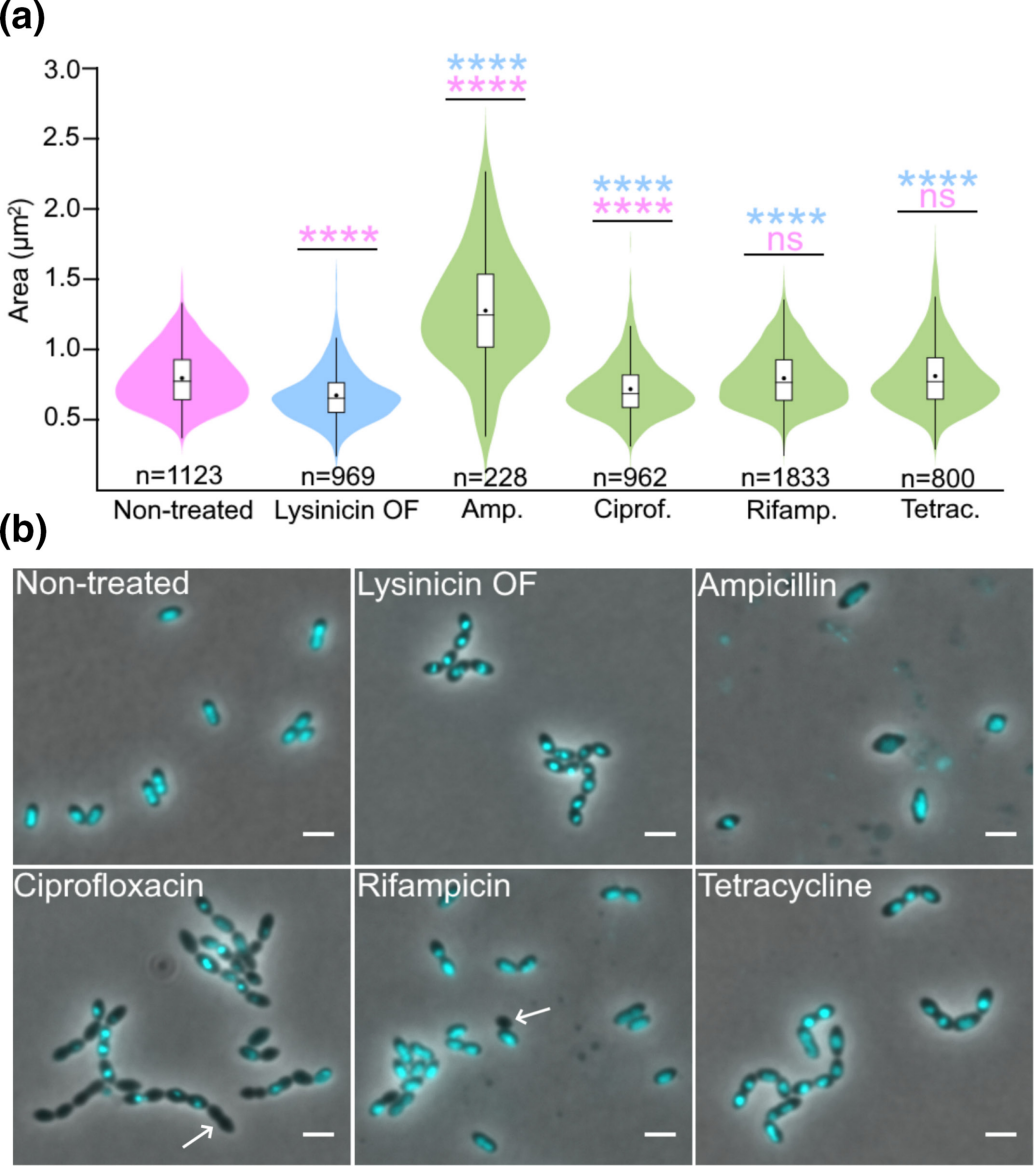
Comparison of the morphology and chromosome distribution in *S. pneumoniae* RH425 treated with lysinicin OF and different antibiotics. (a) Violin plot of the average cell size (µm^2^) after treatment with 1× MIC of the indicated antimicrobials (see Fig. S5) for 4 h at 37 °C. The antibiotics were added at OD_550_=0.1. Compared to non-treated cells (0.79±0.20 µm^2^), ampicillin (1.27±0.40 µm^2^) and ciprofloxacin (0.71±0.19 µm^2^) treatment changed the average cell size, whereas rifampicin (0.79±0.20 µm^2^) and tetracycline (0.80±0.23 µm^2^) did not. Lysinicin OF treatment resulted in smaller cells (0.67±0.18 µm^2^) compared to any other treatment. *P* values were obtained relative to non-treated cells (pink) or lysinicin OF-treated cells (blue) using one-way analysis of variance (ANOVA). ****, *P*<0.00001. (b) The cells were incubated with antibiotics as described for (a). DNA was labelled with DAPI and cells were imaged by phase contrast and fluorescence microscopy. Arrows indicate anucleated cells. Scale bars, 2 µm.

Antibiotics inhibiting cell wall synthesis or DNA replication often induce characteristic changes to bacterial cell size and/or shapes and nucleoid topology, respectively [45–48]. To obtain clues to lysinicin OF’s mode of action, the morphological characteristics of lysinicin OF-treated cells (1× MIC) were compared to those of cells treated with a selection of antibiotics (1× MIC) targeting cell wall synthesis (ampicillin), DNA replication (ciprofloxacin), transcription (rifampicin) and protein synthesis (tetracycline) [49–56]. The nucleoids were also examined by DAPI staining and fluorescence microscopy. Phase contrast imaging did not reveal any dramatic changes to the cell morphology of lysinicin OF-treated cells. However, using the microbeJ analysis tool [12], a reduction in average cell size was observed (from 0.79±0.20 to 0.67±0.18 µm^2^) (Fig. 4a). The cell size was not further reduced by increasing the lysinicin OF concentration to 10× MIC (Fig. S11). Furthermore, lysinicin OF treatment did not cause formation of anucleate cells, but the DAPI signals became clearly more concentrated (Fig. 4b). This suggests that DNA was more densely packed in cells inhibited by lysinicin OF. None of the other antibiotics produced cells with exactly similar characteristics to lysinicin OF treatment. For example, ampicillin treatment resulted in major morphological abnormalities (bloated and elongated cells), while neither rifampicin nor tetracycline resulted in significant cell morphology or cell size changes. We did see a small reduction in average cell size and concentrated DAPI signals for ciprofloxacin. However, ciprofloxacin also produced several anucleated cells, which was not the case for lysinicin OF. Rifampicin was also found to produce some anucleated cells, in line with what has been reported previously in pneumococci [57] (rifampicin is also known to inhibit initiation of replication [58]). Concentrated DAPI signals were observed for tetracycline, but no reduction in cell size. Taken together, none of the antibiotics tested induced identical cellular changes to lysinicin OF, although similar nucleoid topology as for the translation inhibitor tetracycline was seen.

## Discussion

In a screen for anti-pneumococcal compounds among marine bacteria sampled from the Oslo Fjord (Norway), one isolate stood out with strong inhibitory effect. Whole-genome sequencing (GenBank accession number, CP102798) placed it in the genus *Lysinibacillus *, sharing sequence similarities with the species *L. sphaericus* and *L. fusiformis *. The isolate, named *Lysinibacillus* sp. OF-1, produces an anti-pneumococcal compound of proteinaceous nature that we named lysinicin OF. A few previous studies have reported that members of the genus *Lysinibacillus* produce antimicrobials of proteinaceous nature such as heat-labile bacteriocins (inactivated >80 °C) and lipopeptides [59–61]. Since lysinicin OF could resist both heat (100 °C) and polymyxin acylase treatment (cleaves off the peptide part of lipopeptides), we concluded that it most probably is a peptide. Furthermore, BAGEL4 and AntiSMASH mining did not find any gene clusters of known antimicrobials in the *Lysinibacillus* sp. OF-1 genome, suggesting that lysinicin OF is a new antimicrobial peptide. Attempts to identify its mass and amino acid sequence by combining reverse-phase UHPLC and MALDI-TOF MS-MS have unfortunately not succeeded, and we therefore do not know the exact nature of this compound. The explanation for this is that we were unable to obtain a sample of sufficient purity. It is possible that the hydrophobic characteristics of lysinicin OF (binds XAD Amberlite and C18) make it stick to other hydrophobic components in growth medium supernatants that are not removed by the C18 reverse-phase chromatography and acetonitrile gradient elution used here. It would be worth exploring other eluents and solid phases as well as solvent extraction techniques to produce a sample compatible with mass identification and nuclear magnetic resonance (NMR) analysis. Clues about its structure could also be obtained by inactivating genes involved in the biosynthesis of this molecule, e.g. by creating a transposon library of the *Lysinibacillus* sp. OF-1 isolate.

We discovered that lysinicin OF employs the oligo peptide permease Ami in target cells to execute its lethal action. The observation that molecules inhibit growth of *S. pneumoniae* by exploiting the Ami system is not new. In fact, specific peptides derived from ribosomal proteins of Gammaproteobacteria and folate analogues (methotrexate and aminopterin) have been shown to use the Ami system for preventing pneumococcal growth [62, 63]. The exact mechanisms of these peptides and folate analogues, however, remain unknown. Although these oligo peptide permeases are widespread among bacteria [64], lysinicin OF has a narrow target range limited to streptococcal species (except for *S. agalactiae* NCTC8181) and to some extent to *B. subtilis *. The Gram-negatives *P. brenneri* and *E. coli* as well as the Gram-positives *M. smegmatis *, *L. lactis *, *S. aureus* and *E. faecalis* were resistant. Using the pneumococcal AmiC as the reference for a lysinicin OF-sensitive Ami system, sequence comparison of the AmiC homologues in these species with the pneumococcal AmiC (Table S3) showed that a sequence identity <34 % conferred natural tolerance to lysinicin OF. However, there are exceptions, for example *S. bovis* NCTC8177 (26 %), *S. criceti* ATCC19642 (27 %) and *S. mutans* NCTC10449 (28 %). Although having lower homology with AmiC than, for example, the *E. coli* homologue DppB (34 %), these Ami systems probably still have specific structural features and sequence motifs found in pneumococcal Ami, making them recognizable to lysinicin OF.

We showed that cells expressing an intact but inactive Ami system, i.e. Walker B mutated AmiE or AmiF, became completely resistant to lysinicin OF. Combined with the sytox assay and dead/live staining, which showed that lysinicin OF did not interfere with cell membrane integrity, it is plausible that the compound is taken up through the Ami system to hit a cytoplasmic target. If lysinicin OF is indeed a peptide, one would expect that it uses the Ami system for internalization and cells thus become resistant when the Ami system is unable to import peptides. If so, why was a Δ*amiA*, Δ*aliA*, Δ*aliB* triple mutant still sensitive? The Ami system uses either of these lipoproteins to shuttle peptides through the AmiCD channel, and a triple mutant has been shown to have equal oligopeptide transport deficiency to mutants lacking AmiCDE or F [32].

Two alternative explanations are possible. First, lysinicin OF is not taken up by the Ami system, but instead binds the active form of AmiCDEF to induce a conformational change in the complex, e.g. affecting the transmembrane electric potential. It is known that the folic acid derivates aminopterin and methotrexate, which have been used as antineoplastic drugs, inhibit growth of wild-type *S. pneumoniae *, but not in Ami-deficient mutants [62, 65]. Methotrexate was shown to increase the transmembrane electric potential when Ami was intact, but the exact mechanism is unknown [62]. The smallest membrane pores detectable by the sytox assay must allow molecules larger than 278 Da to cross the membrane (MW of Sytox green is 278.329 Da), whereas changes in the transmembrane electric potential would only require opening of an ion channel [66]. A reasonable question is whether lysinicin OF could freeze the AmiCD complex in an open conformation, allowing ions to freely cross the cell membrane. Second, alternatively, lysinicin OF can bypass the requirement of AmiA, AliA and AliB and cross the membrane through the AmiCD channel in its open conformation (AmiEF must be active).

Neither ciprofloxacin, ampicillin, rifampicin, or tetracycline induced identical phenotypic changes to lysinicin OF in *S. pneumoniae *, i.e. reduced cell size and condensed DNA (Fig. 4). Although DNA condensation was also seen for both ciprofloxacin and tetracycline, ciprofloxacin produced several anucleated cells, while tetracycline treatment did not significantly reduce the average cell size. Based on the phenotypic comparison, it seems unlikely that lysinicin OF inhibits cell wall synthesis (ampicillin) or transcription (rifampicin), but rather that its toxic effect somehow can interfere with DNA or protein synthesis. A group of small (<5 kDa) post-translationally modified peptides called class I microcins are known to kill bacteria by inhibiting RNA, DNA and protein synthesis [67]. However, these are produced by enterobacteria (primarily *E. coli *). Microcin-like peptides are yet to be found to be produced by Gram-positive bacteria. Whether lysinicin OF is internalized by sensitive cells, similar to class I microcins, or whether it acts on the outside, somehow transforming the Ami system into a lethal weapon is still unclear at the moment.

Combined with its quick and almost irreversible lethal effect, lysinicin OF could be an interesting molecule to explore for possible therapeutic applications., e.g. prevention of streptococcal host colonization that can lead to skin, soft tissue and mammary gland infections, or within preventative dentistry as an anti-biofilm molecule (mutans group) [68]. However, this study has shown that the antimicrobial potential of lysinicin OF has challenges in terms of resistance development. Full resistance was obtained by inactivation of the Ami system, and Ami-deficient mutants would likely emerge when exposed to lysinicin OF. It has been shown for *S. pneumoniae* that Ami is important for colonization of the host, but not during invasive infection [69]. Therefore, lysinicin OF could have greater potential for preventing streptococcal host colonization rather than for treatment strategies. To further elucidate its therapeutic potential, future investigations should focus on solving the structure and mode of action of this molecule.

## Supplementary Data

Supplementary material 1

## References

[1] IACG (2019). No time to wait: Securing the future from drug-resistant infections.

[2] WHO (2017). Global Antimicrobial Resistance Surveillance System (GLASS) Report Early implementation.

[3] O’Brien KL, Wolfson LJ, Watt JP, Henkle E, Deloria-Knoll M (2009). Burden of disease caused by *Streptococcus pneumoniae* in children younger than 5 years: global estimates. Lancet.

[4] Cherazard R, Epstein M, Doan TL, Salim T, Bharti S (2017). Antimicrobial resistant *Streptococcus pneumoniae*: prevalence, mechanisms, and clinical implications. Am J Ther.

[5] Chewapreecha C, Harris SR, Croucher NJ, Turner C, Marttinen P (2014). Dense genomic sampling identifies highways of pneumococcal recombination. Nat Genet.

[6] Aminov RI (2010). A brief history of the antibiotic era: lessons learned and challenges for the future. Front Microbiol.

[7] Newman DJ, Cragg GM (2007). Natural products as sources of new drugs over the last 25 years. J Nat Prod.

[8] Lacks S, Hotchkiss RD (1960). A study of the genetic material determining an enzyme in pneumococcus. Biochim Biophys Acta.

[9] Higuchi R, Krummel B, Saiki RK (1988). A general method of in vitro preparation and specific mutagenesis of DNA fragments: study of protein and DNA interactions. Nucleic Acids Res.

[10] Sung CK, Li H, Claverys JP, Morrison DA (2001). An rpsL cassette, janus, for gene replacement through negative selection in *Streptococcus pneumoniae*. Appl Environ Microbiol.

[11] Seemann T (2014). Prokka: rapid prokaryotic genome annotation. Bioinformatics.

[12] Ducret A, Quardokus EM, Brun YV (2016). MicrobeJ, a tool for high throughput bacterial cell detection and quantitative analysis. Nat Microbiol.

[13] Laemmli UK (1970). Cleavage of structural proteins during the assembly of the head of bacteriophage T4. Nature.

[14] Stamsås GA, Straume D, Salehian Z, Håvarstein LS (2017). Evidence that pneumococcal WalK is regulated by StkP through protein-protein interaction. Microbiology.

[15] Avram O, Rapoport D, Portugez S, Pupko T (2019). M1CR0B1AL1Z3R-a user-friendly web server for the analysis of large-scale microbial genomics data. Nucleic Acids Res.

[16] van Heel AJ, de Jong A, Song C, Viel JH, Kok J (2018). BAGEL4: a user-friendly web server to thoroughly mine ripps and bacteriocins. Nucleic Acids Res.

[17] Blin K, Shaw S, Kloosterman AM, Charlop-Powers Z, van Wezel GP (2021). AntiSMASH 6.0: improving cluster detection and comparison capabilities. Nucleic Acids Res.

[18] Steller S, Vollenbroich D, Leenders F, Stein T, Conrad B (1999). Structural and functional organization of the fengycin synthetase multienzyme system from *Bacillus subtilis* b213 and A1/3. Chem Biol.

[19] Abriouel H, Franz CMAP, Ben Omar N, Gálvez A (2011). Diversity and applications of *Bacillus* bacteriocins. FEMS Microbiol Rev.

[20] Caulier S, Nannan C, Gillis A, Licciardi F, Bragard C (2019). Overview of the antimicrobial compounds produced by members of the *Bacillus subtilis* group. Front Microbiol.

[21] Yim G, Thaker MN, Koteva K, Wright G (2014). Glycopeptide antibiotic biosynthesis. J Antibiot.

[22] Diep DB, Skaugen M, Salehian Z, Holo H, Nes IF (2007). Common mechanisms of target cell recognition and immunity for class II bacteriocins. Proc Natl Acad Sci.

[23] Kjos M, Oppegård C, Diep DB, Nes IF, Veening J-W (2014). Sensitivity to the two-peptide bacteriocin lactococcin G is dependent on UppP, an enzyme involved in cell-wall synthesis. Mol Microbiol.

[24] Oftedal TF, Ovchinnikov KV, Hestad KA, Goldbeck O, Porcellato D (2021). Ubericin K, a new pore-forming bacteriocin targeting mannose-PTS. Microbiol Spectr.

[25] Ovchinnikov KV, Kristiansen PE, Straume D, Jensen MS, Aleksandrzak-Piekarczyk T (2017). The leaderless bacteriocin enterocin K1 is highly potent against *Enterococcus faecium*: a study on structure, target spectrum and receptor. Front Microbiol.

[26] Zhu L, Zeng J, Wang C, Wang J (2022). Structural basis of pore formation in the mannose phosphotransferase system by pediocin PA-1. Appl Environ Microbiol.

[27] Pérez-Ramos A, Madi-Moussa D, Coucheney F, Drider D (2021). Current knowledge of the mode of action and immunity mechanisms of LAB-bacteriocins. Microorganisms.

[28] Abee T, Klaenhammer TR, Letellier L (1994). Kinetic studies of the action of lactacin F, a bacteriocin produced by *Lactobacillus johnsonii* that forms poration complexes in the cytoplasmic membrane. Appl Environ Microbiol.

[29] Héchard Y, Sahl HG (2002). Mode of action of modified and unmodified bacteriocins from Gram-positive bacteria. Biochimie.

[30] Alloing G, Trombe MC, Claverys JP (1990). The ami locus of the gram-positive bacterium *Streptococcus pneumoniae* is similar to binding protein-dependent transport operons of gram-negative bacteria. Mol Microbiol.

[31] Rees DC, Johnson E, Lewinson O (2009). ABC transporters: the power to change. Nat Rev Mol Cell Biol.

[32] Alloing G, de Philip P, Claverys JP (1994). Three highly homologous membrane-bound lipoproteins participate in oligopeptide transport by the Ami system of the gram-positive *Streptococcus pneumoniae*. J Mol Biol.

[33] Garault P, Le Bars D, Besset C, Monnet V (2002). Three oligopeptide-binding proteins are involved in the oligopeptide transport of *Streptococcus thermophilus*. J Biol Chem.

[34] Davidson AL (2002). Mechanism of coupling of transport to hydrolysis in bacterial ATP-binding cassette transporters. J Bacteriol.

[35] Khan YA, White KI, Brunger AT (2022). The AAA+ superfamily: a review of the structural and mechanistic principles of these molecular machines. Crit Rev Biochem Mol Biol.

[36] Walker JE, Saraste M, Runswick MJ, Gay NJ (1982). Distantly related sequences in the alpha- and beta-subunits of ATP synthase, myosin, kinases and other ATP-requiring enzymes and a common nucleotide binding fold. EMBO J.

[37] Story RM, Steitz TA (1992). Structure of the recA protein-ADP complex. Nature.

[38] Sham LT, Jensen KR, Bruce KE, Winkler ME (2013). Involvement of FtsE ATPase and FtsX extracellular loops 1 and 2 in FtsEX-PcsB complex function in cell division of *Streptococcus pneumoniae* D39. mBio.

[39] Arends SJR, Kustusch RJ, Weiss DS (2009). ATP-binding site lesions in FtsE impair cell division. J Bacteriol.

[40] Breukink E, Wiedemann I, van Kraaij C, Kuipers OP, Sahl HG (1999). Use of the cell wall precursor lipid II by a pore-forming peptide antibiotic. Science.

[41] Ruhr E, Sahl HG (1985). Mode of action of the peptide antibiotic nisin and influence on the membrane potential of whole cells and on cytoplasmic and artificial membrane vesicles. Antimicrob Agents Chemother.

[42] Wiedemann I, Breukink E, van Kraaij C, Kuipers OP, Bierbaum G (2001). Specific binding of nisin to the peptidoglycan precursor lipid II combines pore formation and inhibition of cell wall biosynthesis for potent antibiotic activity. J Biol Chem.

[43] Schmitt MA, Weisblum B, Gellman SH (2007). Interplay among folding, sequence, and lipophilicity in the antibacterial and hemolytic activities of alpha/beta-peptides. J Am Chem Soc.

[44] Wang G, Li X, Wang Z (2016). APD3: the antimicrobial peptide database as a tool for research and education. Nucleic Acids Res.

[45] Lorian V, Atkinson B (1975). Abnormal forms of bacteria produced by antibiotics. Am J Clin Pathol.

[46] Georgopapadakou NH, Bertasso A (1991). Effects of quinolones on nucleoid segregation in *Escherichia coli*. Antimicrob Agents Chemother.

[47] Rajendram M, Hurley KA, Foss MH, Thornton KM, Moore JT (2014). Gyramides prevent bacterial growth by inhibiting DNA gyrase and altering chromosome topology. ACS Chem Biol.

[48] Hawkey PM (2003). Mechanisms of quinolone action and microbial response. J Antimicrob Chemother.

[49] Hartmann G, Honikel KO, Knüsel F, Nüesch J (1967). The specific inhibition of the DNA-directed RNA synthesis by rifamycin. Biochim Biophys Acta.

[50] Wehrli W, Knüsel F, Schmid K, Staehelin M (1968). Interaction of rifamycin with bacterial RNA polymerase. Proc Natl Acad Sci.

[51] Connamacher RH, Mandel HG (1965). Binding of tetracycline to the 30S ribosomes and to polyuridylic acid. Biochem Biophys Res Commun.

[52] Gale EF, Folkes JP (1953). The assimilation of amino-acids by bacteria. XV. Actions of antibiotics on nucleic acid and protein synthesis in S*taphylococcus aureus*. Biochem J.

[53] Izaki K, Matsuhashi M, Strominger JL (1966). Glycopeptide transpeptidase and D-alanine carboxypeptidase: penicillin-sensitive enzymatic reactions. Proc Natl Acad Sci.

[54] Lawrence PJ, Strominger JL (1970). Biosynthesis of the peptidoglycan of bacterial cell walls. XV. The binding of radioactive penicillin to the particulate enzyme preparation of *Bacillus subtilis* and its reversal with hydroxylamine or thiols. J Biol Chem.

[55] Sugino A, Peebles CL, Kreuzer KN, Cozzarelli NR (1977). Mechanism of action of nalidixic acid: purification of *Escherichia coli* nalA gene product and its relationship to DNA gyrase and a novel nicking-closing enzyme. Proc Natl Acad Sci.

[56] Emmerson AM, Jones AM (2003). The quinolones: decades of development and use. J Antimicrob Chemother.

[57] Kjos M, Veening JW (2014). Tracking of chromosome dynamics in live *Streptococcus pneumoniae* reveals that transcription promotes chromosome segregation. Mol Microbiol.

[58] von Meyenburg K, Hansen FG, Riise E, Bergmans HE, Meijer M (1979). Origin of replication, oriC, of the *Escherichia coli* K12 chromosome: genetic mapping and minichromosome replication. Cold Spring Harb Symp Quant Biol.

[59] Ahmad V, Iqbal A, Haseeb M, Khan MS (2014). Antimicrobial potential of bacteriocin producing *Lysinibacillus* jx416856 against foodborne bacterial and fungal pathogens, isolated from fruits and vegetable waste. Anaerobe.

[60] Akintayo SO, Treinen C, Vahidinasab M, Pfannstiel J, Bertsche U (2022). Exploration of surfactin production by newly isolated *Bacillus* and *Lysinibacillus* strains from food-related sources. Lett Appl Microbiol.

[61] Satapute P, Jogaiah S (2022). A biogenic microbial biosurfactin that degrades difenoconazole fungicide with potential antimicrobial and oil displacement properties. Chemosphere.

[62] Trombe MC (1984). Alteration of *Streptococcus pneumoniae* membrane properties by the folate analog methotrexate. J Bacteriol.

[63] Nasher F, Aguilar F, Aebi S, Hermans PWM, Heller M (2018). Peptide ligands of AmiA, AliA, and AliB proteins determine pneumococcal phenotype. Front Microbiol.

[64] Monnet V (2003). Bacterial oligopeptide-binding proteins. Cell Mol Life Sci.

[65] Sicard AM (1964). A new synthetic medium for *Diplococcus pneumoniae*, and its use for the study of reciprocal transformations at the Amia Locus. Genetics.

[66] Martinac B, Saimi Y, Kung C (2008). Ion channels in microbes. Physiol Rev.

[67] Duquesne S, Destoumieux-Garzón D, Peduzzi J, Rebuffat S (2007). Microcins, gene-encoded antibacterial peptides from enterobacteria. Nat Prod Rep.

[68] Oda Y, Hayashi F, Okada M (2015). Longitudinal study of dental caries incidence associated with *Streptococcus mutans* and *Streptococcus sobrinus* in patients with intellectual disabilities. BMC Oral Health.

[69] Kerr AR, Adrian PV, Estevão S, de Groot R, Alloing G (2004). The Ami-AliA/AliB permease of *Streptococcus pneumoniae* is involved in nasopharyngeal colonization but not in invasive disease. Infect Immun.

[70] Slager J, Aprianto R, Veening JW (2018). Deep genome annotation of the opportunistic human pathogen *Streptococcus pneumoniae* D39. Nucleic Acids Res.

