# Supplementary material for: A novel proteinaceous molecule produced by Lysinibacillus sp. OF-1 depends on the Ami oligopeptide transporter to kill Streptococcus pneumoniae
Source: Microbiology (Reading). 2023 Mar 7;169(3):001313. doi: 10.1099/mic.0.001313 (PMC10191383; doi:10.1099/mic.0.001313)
Supplement: Supplementary material 1 [file mic-169-1313-s001.pdf]

Supplemental material for the article entitled: A novel proteinaceous molecule produced by *Lysinibacillus* sp. OF-1 depends on the Ami oligopeptide transporter to kill *Streptococcus pneumoniae*.

Ingvild Hals Hauge[1], Vilde Sandegren[1], Anja Ruud Winther[1], Cathrine Arnason Bøe[2], Zhian Salehian[1], Leiv Sigve Håvarstein[1], Morten Kjos[1] and Daniel Straume[1]*.

[1]Faculty of Chemistry, Biotechnology and Food Science, Norwegian University of Life Sciences, 1430 Ås, Norway.

[2]Norwegian Veterinary Institute, Department of Molecular Biology, 1433 Ås, Norway.

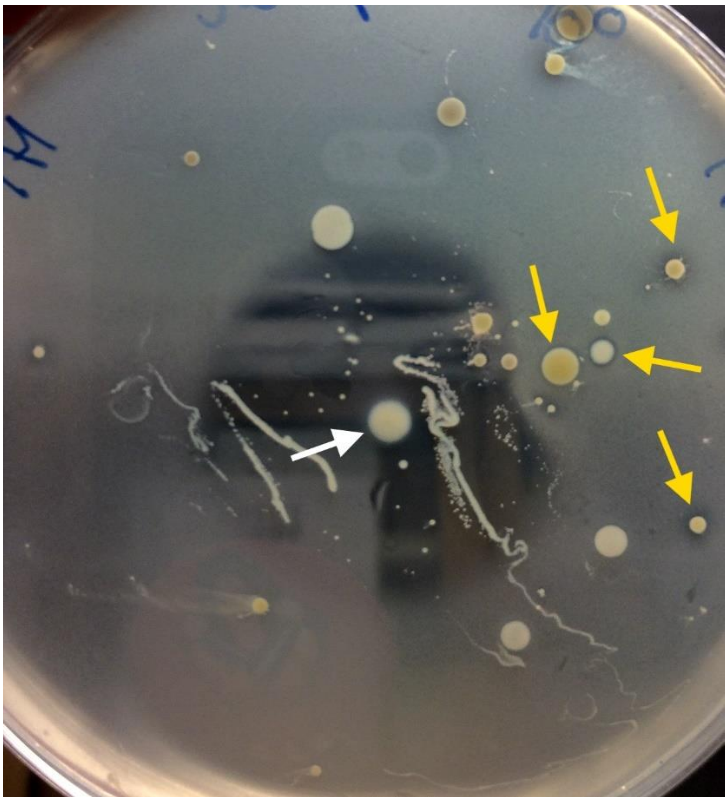

Fig. S1. Initial soft-agar overlay showing growth inhibition of *S. pneumoniae* RH425 by colonies isolated from a rock at the shore in the Oslo Fjord. The yellow arrows indicate colonies surrounded by smaller inhibition zones, while the white arrow indicates a colony identified as a *Lysinibacillus* species with strong inhibition of *S. pneumoniae*.

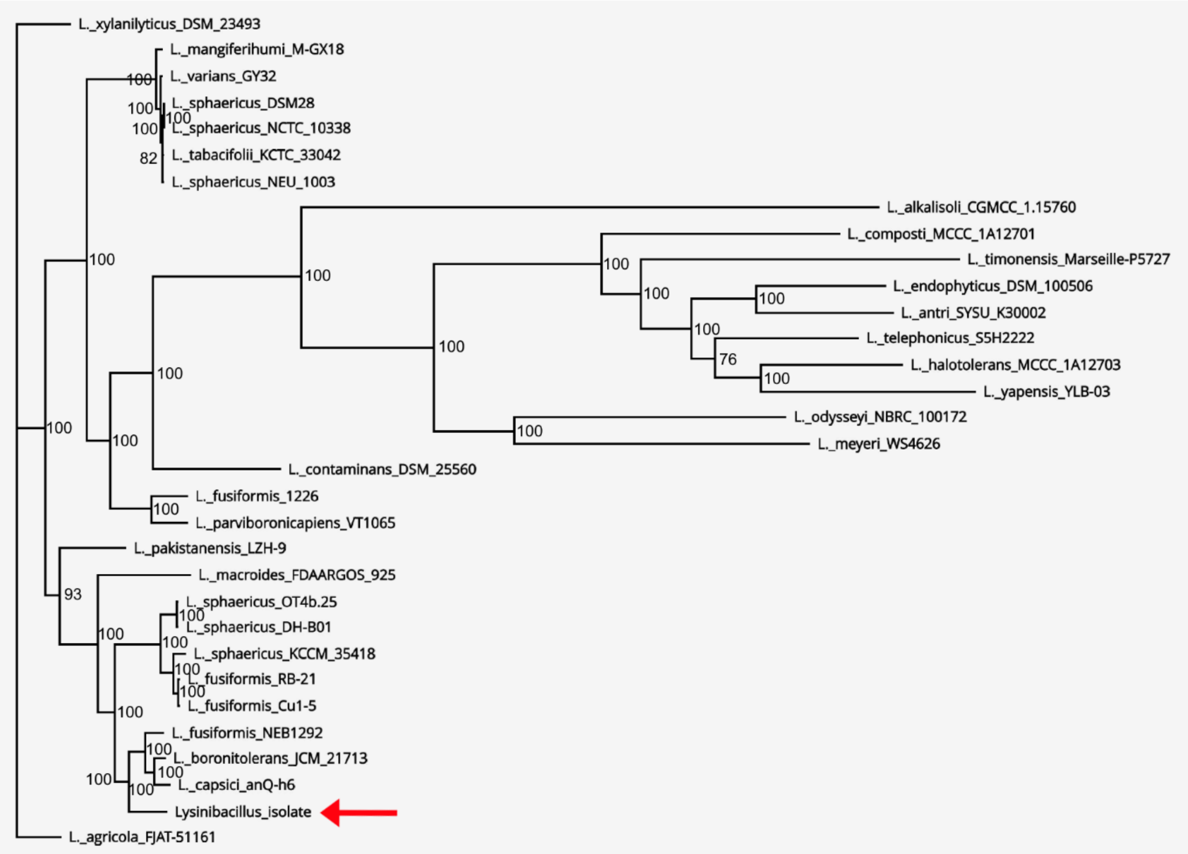

Fig. S2. Phylogram showing the evolutionary relationship of 31 lysinibacilli, including all 23 known species in this genus. *Lysinibacillus* sp. OF-1 is placed (red arrow) in a clade together with *L. fusiformis*, *L. boronitolerans* and *L. capsici*.

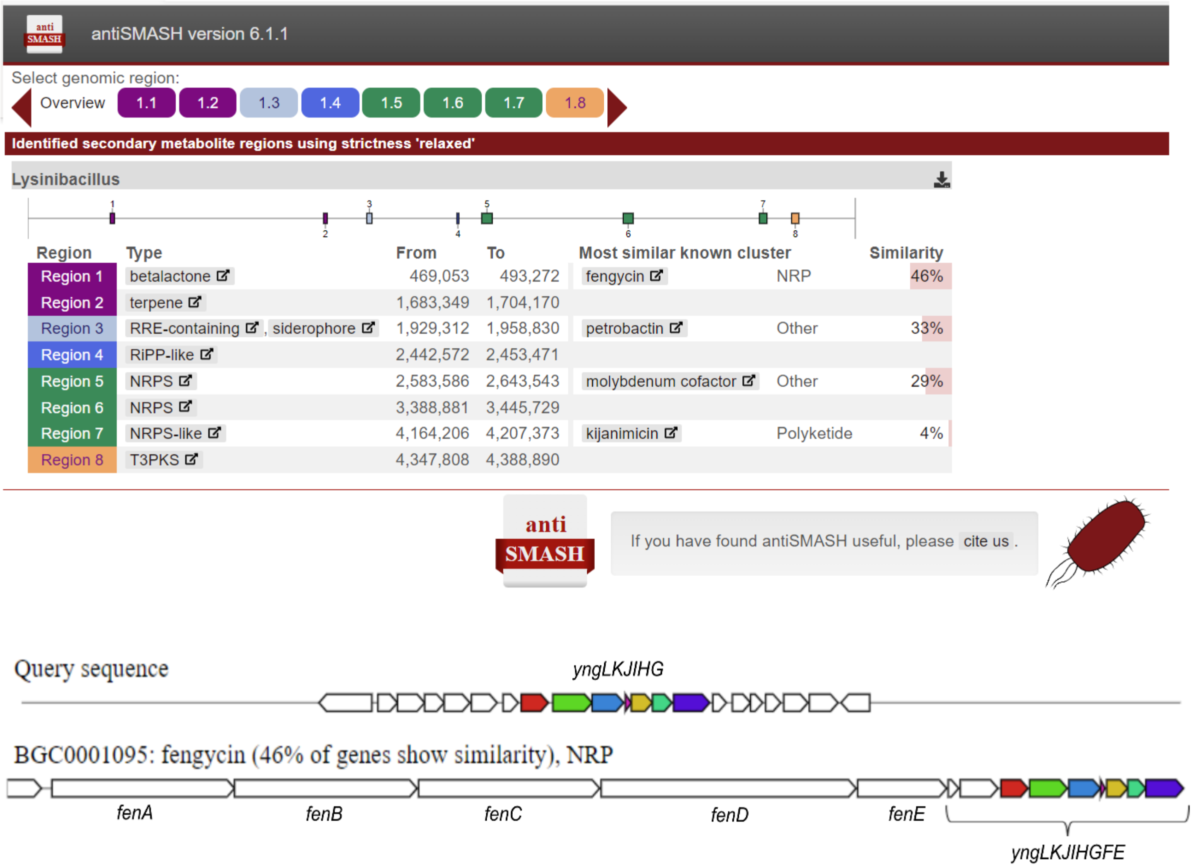

Fig. S3. antiSMASH output of the *Lysinibacillus* sp. OF-1 genome analysis. A 46% similarity (percentage of genes) to the fengycin biosynthesis cluster in *B. velezensis* FZB42 was found. The *Lysinibacillus* sp. OF-1 genome does not have the fengycin synthetase genes *fenABCDE* but has the *yngLKJIHG* genes, which are often found associated with fengycin synthetase genes (1). The exact functions of the *yng* genes are not known, but they have been suggested to be involved in lipid catabolism, leucine degradation and/or sporulation (2, 3).

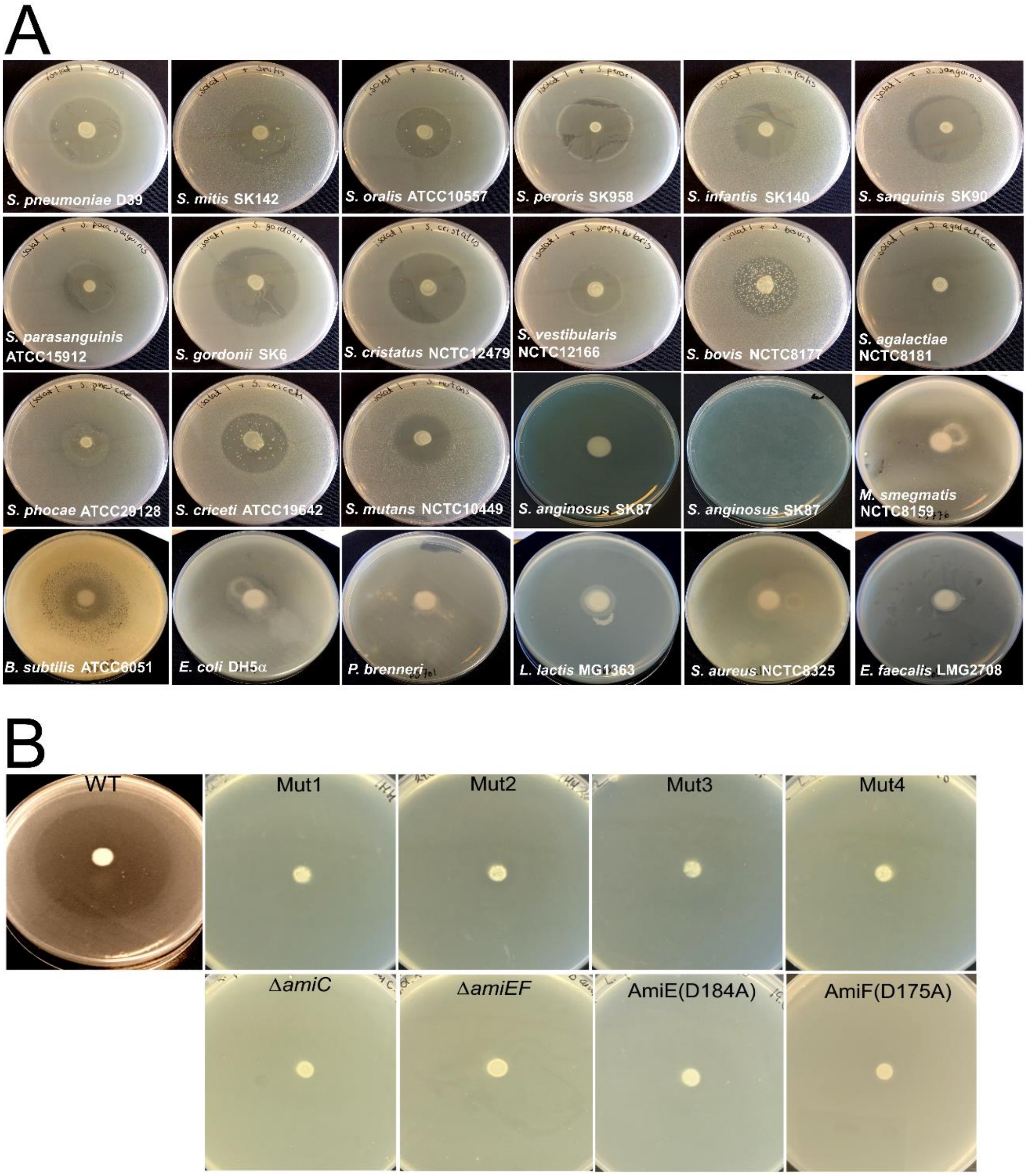

Fig. S4. A. Soft-agar overlays placed on top of two days old *Lysinibacillus* sp. OF-1 spots. Bacteria added to the soft-agar are indicated. *S. criceti* and *S. bovis*, which grew in aggregates in liquid culture, contained several colonies of resistant cells in their inhibition zones, suggesting that cell aggregation could provide protection against lysinicin OF. For some other overlays, e.g., *S. peroris*, *S. gordonii*, *S. sanguinis* and *S. phocae*, *Lysinibacillus* sp. OF-1 had started to migrate into the inhibition zones, which appeared as a grey coating on top of the soft-agar. The inhibition zone of *S. anginosus* covered nearly the whole agar plate, and an overlay without the *Lysinicibacillus* sp. OF-1 spot was included as control of *S. anginosus* growth. **B.** In the upper panels, *S. pneumoniae* mutant 1-4 (spontaneous lysinicin OF resistant mutants) were used as indicator strains, while the lower panels represent overlays containing mutants Δ*amiC*, Δ*amiEF*, AmiE^{D184A} and AmiF^{D175A}. All pneumococcal mutants were resistant to Lysinicin OF as no inhibition zones were observed around the *Lysinibacillus* sp. OF-1 spots. An overlay containing wild type was included to show a typical inhibition zone.

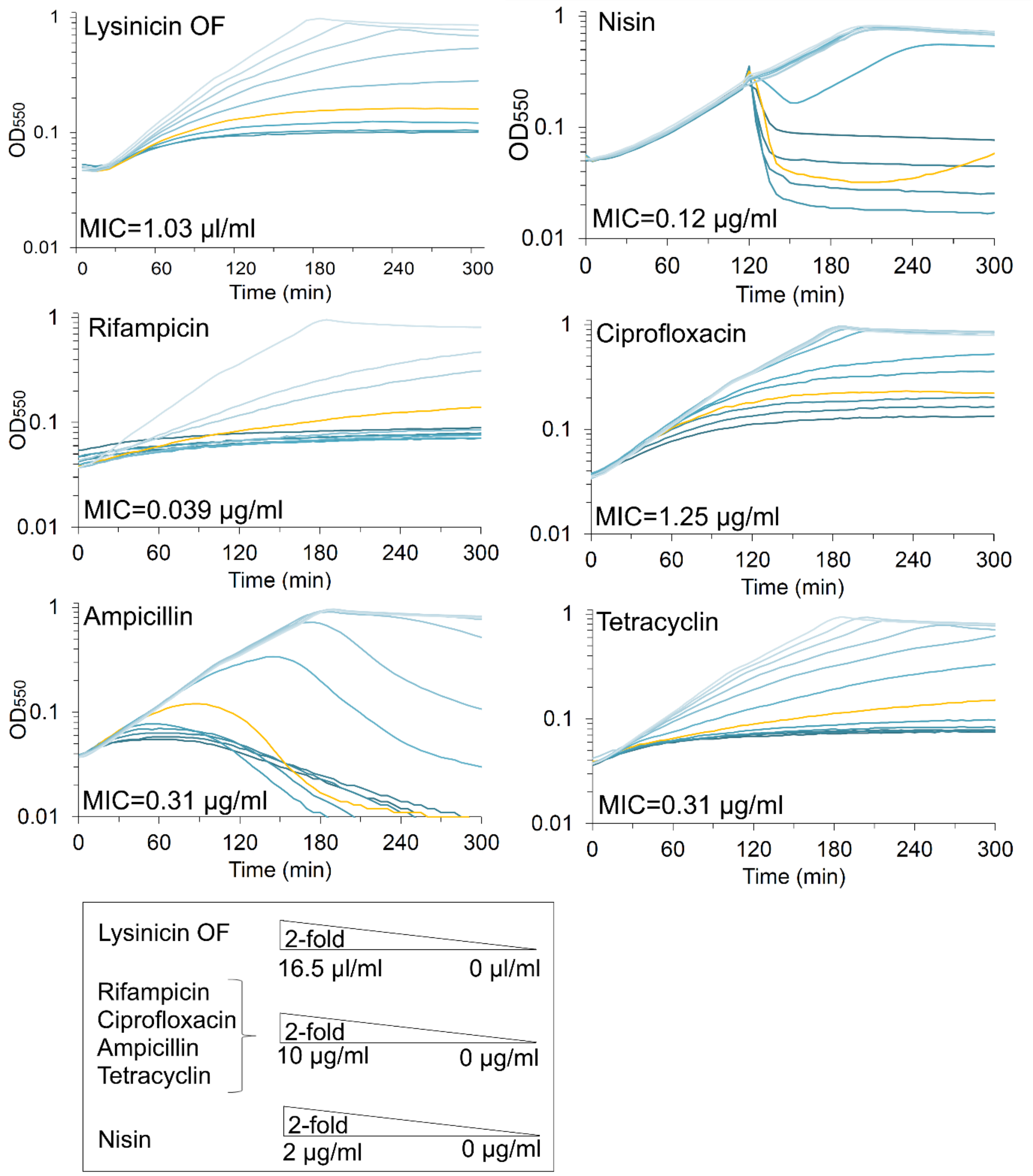

Fig. S5. Estimations of MIC values. *S. pneumoniae* RH425 was grown in the presence of two-fold dilution series of lysinicin OF, rifampicin, ciprofloxacin, ampicillin, tetracycline and nisin. The yellow growth curves were defined as MIC values (indicated in each panel). The dilution series used for each antimicrobial are shown at the bottom left.

```
AmiE      MTKEKNVILTARDIVVEFDVRDKVLTAIRGVSLELVEGEVLALVGESGSGKSVLTKTFTG      60
AmiF      ---MSEKLVEIKDLEISFGEGSKKFVAVKNANFFINKGETFSLVGESGSGKTTIGRAIIG      57
DppD      ----MSIIIRVEDLRAVYLVREGTIKAADGISLDILENSVTAIVGESASGKSTIIEAMTK      56
               . ::  .*:   :   .  : *  . .: : :...  ::****.***:.: .::

AmiE      MLEENGRIAQGSIDYRGQDLTALSSHKDWEQIRGAKIATIFQDPMTSLDPIKTIGSQITE      120
AmiF      LND----TSNGDIIFDGQKINGKKSREQAA-ELIRRIQMIFQDPAASLNERATVDYIISE      112
DppD      TLPPNGRILSGRVLYKGKDLLTMREEE-LRKIRWKEIALVPQAAQQSLNPTMKVIEHFKD      115
                   .* : : *:.:    ..:        .*  : *    **:   .:   :.:

AmiE      VIVKHQGKTA-KEAKELAIDYMNKVGIPDADRRFNEYPFQYSGGMRQRIVIAIALACRPD      179
AmiF      GLYNHRLFKDEEERKEKVQSIIREVGLL--AEHLTRYPHEFSGGQRQRIGIARALVMQPD      170
DppD      TVEAHGVRWSHSELIEKASEKL-RMVRLNPEAVLNSYPLQLSGGMKQRVLIALALLLDPV      174
           :  *      .*  *  . . : .:        :. **  : *** :**: ** **   *

AmiE      VLICDEPTTALDVTIQAQIIDLLKSLQNEYHFTTIFITHDLGVVASIADKVAVMYAGEIV      239
AmiF      FVIADEPISALDVSVRAQVLNLLKKFQKELGLTYLFIAHDLSVVRFISDRIAVIYKGVIV      230
DppD      VLILDEPTSALDVLTQAHIIQLLKELKKMLKITLIFVTHDIAVAAELADKVAVIYGGNLV      234
          .:* *** :****  :*:::***.:::    :* :*::**:.*.  ::*::**:* * :*

AmiE      EYGTVEEVFYDPRHPYTWSLLSSLPQLADDKGDLYSIPGTPPSLYTDLKGDAFALRSDYA      299
AmiF      EVAETEELFNNPIHPYTQALLSAVPIPDPILERKKVLKVYDPS------QHDYET-----      279
DppD      EYNSTFQIFKNPLHPYTRGLINSIMAVNADMSKVKPIPGDPPSLLNPPSGCRFHPRCEYA      294
          *   . ::* :* **** .*:.::            :    **         :

AmiE      MQIDFEQKAPQFSVSET--HWAKTW---LLHEDAPKVEKPAVIANLHDKIREKMGFAHLA      354
AmiF      ------DKPSMVEIRPGHYVWANQAELARYQKGLN-------------------------      308
DppD      MEICKKEKPKWIRLDGE--AHVACH---LYEEGRPLKLE---------------------      328
                :*   . :       .        .:.

AmiE      D      355
AmiF      -      308
DppD      -      328
```

Fig. S6. Multiple sequence alignment of AmiE, AmiF and DppD (PDB 4FWI). The conserved Walker A and Walker B motifs are shown in green and yellow, respectively. The aspartic acid residue involved in coordination of a Mg^{2+} ion is boxed (D184 in AmiE and D175 in AmiF).

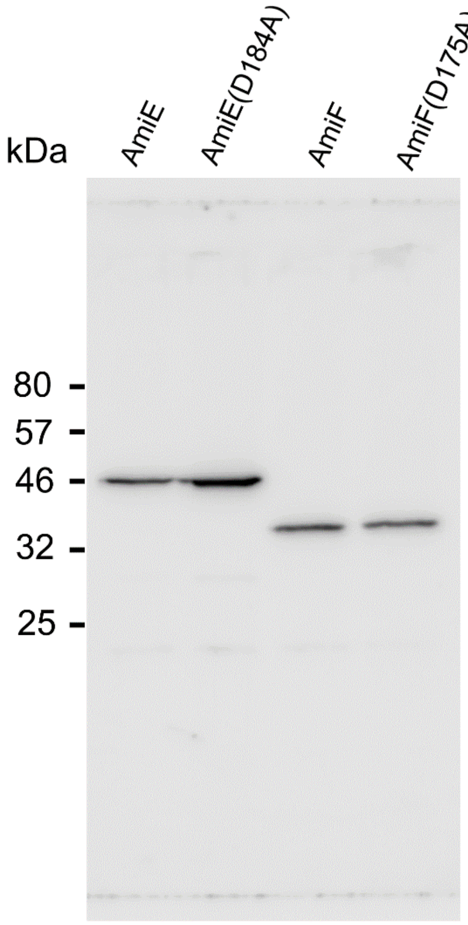

Fig. S7. The D184A and D175A mutations did not reduce the stability of AmiE and AmiF in *S. pneumoniae*. Immunodetection of C-terminally Flag-tagged AmiE, AmiF and their D184A and D175A counterparts in whole cell extracts. The genes were expressed from their native loci. Exponentially growing cells from 5 ml cultures were collected at OD_{550} = 0.25 and resuspended in 100 µl SDS sample buffer. The samples were heated at 95°C for 10 min before 15 µl samples were separated in a 12% SDS-PAG and subsequent electroblotting and immunodetection.

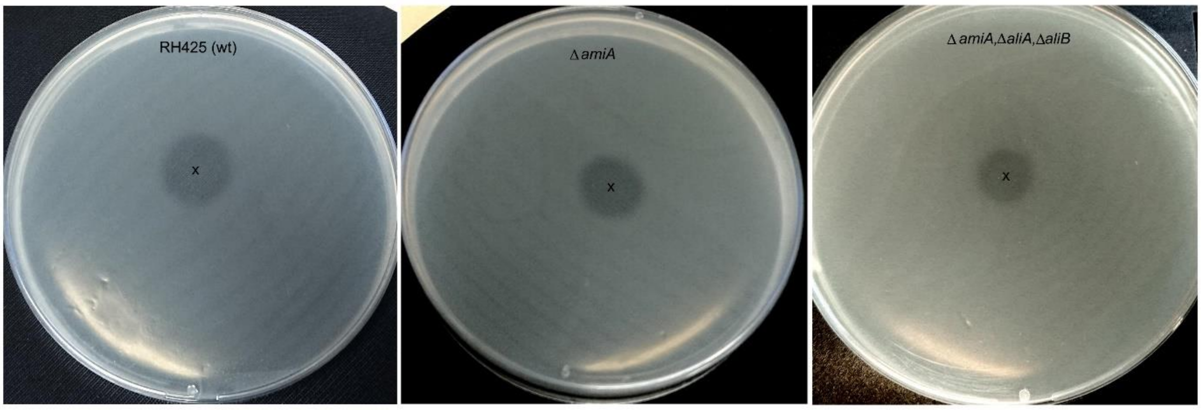

Fig. S8. The Δ*amiA* and Δ*amiA*, Δ*aliA*, Δ*aliB* triple mutants were sensitive to lysinicin OF. Three µl of lysinicin OF was spotted on top of a soft-agar overlay (marked with an x) containing either *S. pneumoniae* RH425, a Δ*amiA* or a Δ*amiA*, Δ*aliA*, Δ*aliB* mutant. Growth inhibition is seen as clear zones in the soft-agar.

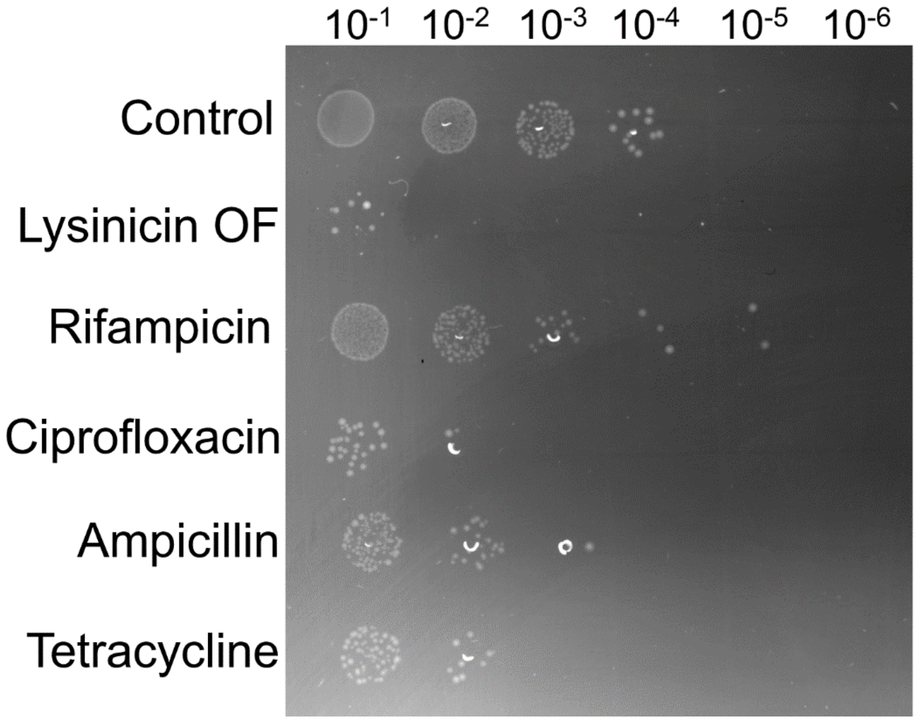

Fig. S9. Spot-assay comparing the survival of lysinicin OF treated *S. pneumoniae* with other antibiotic treatments. Cells were treated for 30 min with 10xMIC of each antimicrobial before antibiotic removal and OD adjustment. The different antibiotics are indicated on the figure left, and the dilution of cell culture on the top. Three µl of each dilution were spotted on TH-agar.

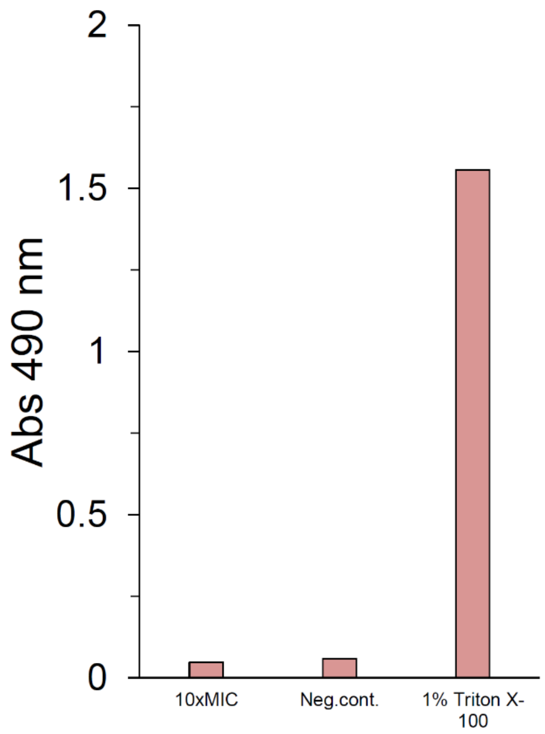

Fig. S10. Lysinicin OF did not cause hemolysis of sheep blood. The absorption at 490 nm in the supernatants of blood samples treated with 10xMIC of Lysinicin OF were compared with supernatants of non-treated blood and blood treated with 1% (v/v) Triton X-100.

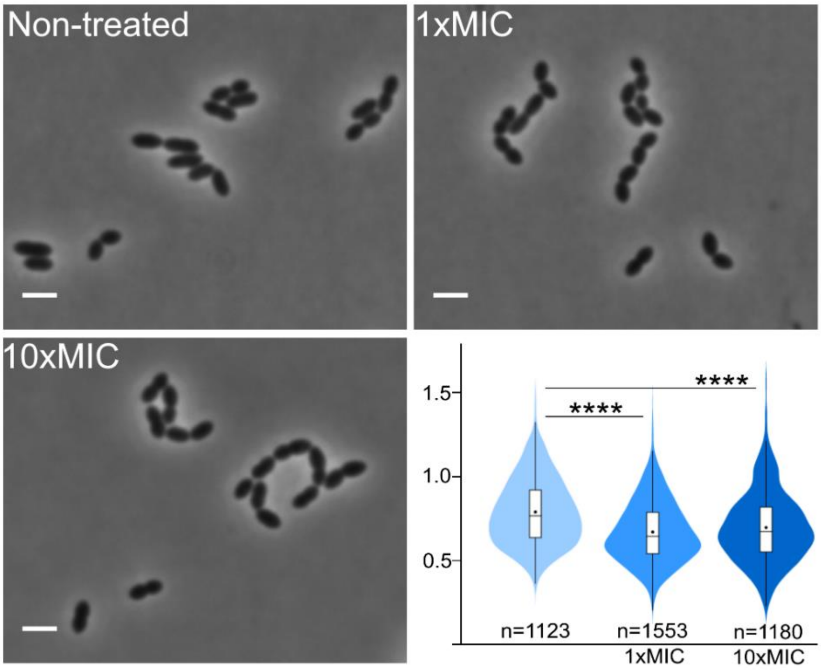

Fig. S11. Concentrations of lysinicin OF corresponding to 1xMIC (1 µl/ml) and 10xMIC (10 µl/ml) was added to *S. pneumoniae* at OD_{550} = 0.1. After four hours at 37°C, cells were imaged by phase contrast microscopy, and the average cell sizes (area in µm^2) were estimated using MicrobeJ, here represented as violin plots. Average cell size ranged from 0.79 ± 0.20 µm^2 (non-treated) to 0.67 ± 0.18 µm^2 (1xMIC) and 0.70 ± 0.21 µm^2 (10xMIC). P values were obtained relative to non-treated cells using one-way analysis of variance (ANOVA). ****, P < 0.00001. Scale bars are 2 µm.

Table S1. Bacterial species used in this study.

***S. pneumoniae* strains**	Relevant characteristics	Source
RH14	R6 derivative, Δ*comA*::*ermAM*, Δ*lytA*::kan; Ery^r, Kan^r	(4)
RH425	R6 derivative, but Δ*comA*::*ermAM, rpsL1*; Ery^r, Sm^r	(5)
IHH21	RH425, but Δ*amiC*::janus, Ery^r, Kan^r	This study
IHH22	RH425, but Δ*amiEF*::janus, Ery^r, Kan^r	This study
IHH23	RH425, but *amiE*(D184A); Ery^r, Sm^r	This study
aw495	RH425, but *amiF*(D190A); Ery^r, Sm^r	This study
VS14	RH425, but *amiE-Flag*; Ery^r, Sm^r	This study
VS15	RH425, but *amiE(D184)-Flag*; Ery^r, Sm^r	This study
VS16	RH425, but *amiF-Flag*; Ery^r, Sm^r	This study
VS17	RH425, but *amiF(D175A)*-Flag; Ery^r, Sm^r	This study
ds1024	RH425, but Δ*amiA*::*aad9*; Ery^r, Sm^r, Spc^r	This study
ds1030	RH425, but Δ*amiA*::*aad9,* Δ*aliA*::janus, Δ*aliB*::*cat*; Ery^r, Spc^r, Kan^r, Cm^r	This study
mut1	RH425, but C→T at position 1676261, truncated AmiC; Ery^r, Sm^r	This study
mut2	RH425, but G→A at position 1673543, truncated AmiE; Ery^r, Sm^r	This study
mut3	RH425, but deletion from position 1672934-1674011, truncated AmiE and Δ*amiF*; Ery^r, Sm^r	This study
mut4	RH425, but G→T at position 1673313, truncated AmiF; Ery^r, Sm^r	This study
D39	Wild type	(6)
**Other streptococcal species**[a]		
S. mitis SK142	Wild type	Lab stock
S. oralis ATCC10557	Wild type	Lab stock
S. peroris SK958	Wild type	Lab stock
S. infantis SK140	Wild type	Lab stock
S. sanguinis SK90	Wild type	Lab stock
S. parasanguinis ATCC15912	Wild type	Lab stock
S. gordonii SK6	Wild type	Lab stock
S. cristatus NCTC12479	Wild type	Lab stock
S. vestibularis NCTC 12166	Wild type	Lab stock
S. bovis NCTC8177	Wild type	Lab stock
S. agalactiae NCTC8181	Wild type	Lab stock
S. phocae ATCC29128	Wild type	Lab stock
S. criceti ATCC19642	Wild type	Lab stock
S. mutans NCTC10449	Wild type	Lab stock

S. angonisus SK87	Wild type	Lab stock
**Other bacterial species**		
B. subtilis ATCC6051	Wild type	ATCC
M. smegmatis NCTC8159	Wild type	UKHSA
E. coli DH5a	Cloning host	Invitrogen
Pseudomonas brenneri, Norwegian isolate	Wild type	Lab stock, this study
L. lactis MG1363	Wild type	Lab stock
S. aureus NCTC8325	Wild type	Lab stock
E. faecalis LMG2708	Wild type	Lab stock
Lysinibacillus sp. OF-1		This study

[a]Streptococcal lab stocks were kindly provided by Prof. Mogens Kilian.

Table S2. Oligoes used for PCR.

Oligo name	Sequence (5'→3')	Source
Primers used for 16S rDNA amplification		
11F	TAACACATGCAAGTCGAACG	(7)
1492R	GGTTACCTTGTTACGACTT	(8)
Primers used for amplifying the Janus cassette		
Kan484.F	GTTTGATTTTTAATGGATAATGTG	(9)
RpsL41.R	CTTTCCTTATGCTTTTGGAC	(9)
Construction of a Δ*amiC*::janus cassette (used in combination with Kan484.F and RpsL41.R)		
IHH3	AATATCTATTACACACAATCAGG	This study
IHH4	CACATTATCCATTAAAAATCAAACCATGGAGAGAAAGTTCTATTAG	This study
IHH5	GTCCAAAAGCATAAGGAAAGGGTAAAATGTTGATTGACTCTG	This study
IHH6	GGACAAGGATACCAAGACAAGG	This study
Construction of a Δ*amiEF*::janus cassette (used in combination with Kan484.F and RpsL41.R)		
IHH7	TCTAATAACTCTATGGTCGTTG	This study
IHH8	CACATTATCCATTAAAAATCAAACTTCTACTCCTATCTATGTGTAC	This study
IHH9	GTCCAAAAGCATAAGGAAAGTGGTCGTGCTATCATCGGTC	This study
IHH10	TTAGTCCTTTTTGATAACGTGC	This study

Construction of an *amiE* (D184A) cassette (IHH11 and IHH12 were used in combination with IHH7 and IHH 10, respectively).		
IHH11	AGCACAGATCAAGACATCAGG	This study
IHH12	CCTGATGTCTTGATCTGTGCTGAGCCAACAACTGCCTTG G	This study
Construction of an *amiF* (D190A) cassette (IHH13 and IHH14 were used in combination with IHH7 and IHH10, respectively).		
IHH13	AGCTGCAATAACAAAGTCTGGT	This study
IHH14	ACCAGACTTTGTTATTGCAGCTGAGCCAATTTCAGCCTT GGAC	This study
Primers used for C-terminal flag tagging of AmiE (VS15 and VS16 were used in combination with IHH7 and IHH10, respectively)		
VS15	GATTATAAAGATGATGATGATAAATAGGAGGAAGGAA ATGTCTGAAAAATTAG	This study
VS16	CTATTTATCATCATCATCTTTATAATCGTCAGCCAGATG GGCAAATCC	This study
Primers used for C-terminal flag tagging of AmiF (VS17 and VS18 were used in combination with IHH7 and IHH10, respectively)		
VS17	GATTATAAAGATGATGATGATAAATAATAATGGTTTTAT AATTTCCATGTC	This study
VS18	TTATTTATCATCATCATCTTTATAATCGTTTAGTCCTTTT TGATAACGTGC	This study
Construction of Δ*amiA*::*aad9*		
aad9 F	GTGAGGAGGATATATTTGAATAC	This study
aad9 R	TTATAATTTTTTTAATCTGTTATTTAAATAG	This study
VS1	CTTTATATTGATACGATTCTGAG	This study
VS4	GTGTTCTTGAAACGAGCCATG	This study
ds794	GTATTCAAATATATCCTCCTCACCAACCCTTTCAACAAG AATGG	This study
ds795	ATTTAAATAACAGATTAAAAAAATTATAACTCAAATCA ATGGTAAAGATGG	This study
Construction of Δ*aliA*::janus (used in combination with Kan484.F and RpsL41.R)		
Ds802	AAGGCGACGCTAAGCTTGG	This study
Ds803	CACATTATCCATTAAAAATCAAACCTCTCCATTATAGAC TCTTTTC	This study
Ds804	GTCCAAAAGCATAAGGAAAGAAAACATGTGAAATAACT GTTGC	This study
Ds805	GCAGCAACACGACTACCTC	This study
Construction of Δ*aliB*::*cat*		
Ds806	TAAGCGTCTCTTGGTTGATAC	This study
Ds807	CCTTTTTTAAAAGTCAATATTACTGTTCCAGAACCTCCT GC	This study
Ds808	GCCTAATGACTGGCTTTTATAAAATCTAATTGTAGATAA GTTTGTG	This study
Ds809	TAGGATTAAGTAATTGAAAGAGG	This study
Cam F	CAGTAATATTGACTTTTAAAAAAGG	This study
Cam R	TTATAAAAGCCAGTCATTAGGC	This study
Primers used for sequencing of the *ami*-locus.		
ds679	TCACTGTAGTCTTTGACACTTC	This study

ds680	CTGAATGAAGAATTCGAAACATC	This study
ds682	AATTGATTTTCAAGCAGGATCC	This study
ds683	TTGGTTCAGCCATGGCTCG	This study
ds684	GATTTCAATGATGTCAGCAAGG	This study
ds685	GTGGAATTTGACGTTCGTGAC	This study
ds686	GATGCTTTTGCCTTGCGTTC	This study
ds687	GCCTTGGACGTTTCTGTACG	This study
ds688	GCTCATACAACAGGATAGTCG	This study
ds691	CCCAAAGTCCAACCATGACC	This study

Table S3. Different bacteria's sensitivity to lysinicin OF and identity of their AmiC homologues relative to the R6 AmiC

Species	Sensitive	% identity to R6 AmiC	Source
S. pneumoniae R6	Yes		J.P. Claverys
S. pneumoniae D39	Yes	100	(6)
S. mitis SK142	Yes	74 (ATCC 903)[a]	M. Kilian
S. oralis ATCC10557	Yes	92	M. Kilian
S. peroris SK958	Yes	92	M. Kilian
S. infantis SK140	Yes	93	M. Kilian
S. sanguinis SK90	Yes	81 (ATCC 29667)[a]	M. Kilian
S. parasanguinis ATCC15912	Yes	74	M. Kilian
S. gordonii SK6	Yes	83 (challis)[a]	M. Kilian
S. cristatus NCTC12479	Yes	80	M. Kilian
S. vestibularis NCTC 12166	Yes	77	M. Kilian
S. bovis NCTC8177	Yes	26	M. Kilian
S. agalactiae NCTC8181	No	27	M. Kilian
S. phocae ATCC29128	Yes	65	M. Kilian
S. criceti ATCC19642	Yes	27	M. Kilian
S. mutans NCTC10449	Yes	28	M. Kilian
S. anginosus SK87	Yes	83 (ATCC 12395)[a]	M. Kilian
B. subtilis ATCC6051	Moderate	28	ATCC
M. smegmatis NCTC8159	No	28	UKHSA
E. coli DH5a	No	34	Invitrogen
Pseudomonas brenneri	No	29 (FH4)[a]	Lab stock, This study
L. lactis MG1363	No	33	Lab stock
S. aureus NCTC8325	No	30	Lab stock
E. faecalis LMG2708	No	34 (ATCC 29212)[a]	Lab stock

[a] Indicates the strain used to for comparison with the pneumococcal AmiC sequence.

References.

1. Koumoutsi A, Chen XH, Henne A, Liesegang H, Hitzeroth G, Franke P, et al. Structural and functional characterization of gene clusters directing nonribosomal synthesis of bioactive cyclic lipopeptides in *Bacillus amyloliquefaciens* strain FZB42. J Bacteriol. 2004;186(4):1084-96.
2. Hsiao TL, Revelles O, Chen L, Sauer U, Vitkup D. Automatic policing of biochemical annotations using genomic correlations. Nat Chem Biol. 2010;6(1):34-40.
3. Eichenberger P, Fujita M, Jensen ST, Conlon EM, Rudner DZ, Wang ST, et al. The program of gene transcription for a single differentiating cell type during sporulation in *Bacillus subtilis*. PLoS Biol. 2004;2(10):e328.
4. Eldholm V, Johnsborg O, Haugen K, Ohnstad HS, Håvarstein LS. Fratricide in *Streptococcus pneumoniae*: contributions and role of the cell wall hydrolases CbpD, LytA and LytC. Microbiology (Reading, England). 2009;155(Pt 7):2223-34.
5. Johnsborg O, Håvarstein LS. Pneumococcal LytR, a protein from the LytR-CpsA-Psr family, is essential for normal septum formation in *Streptococcus pneumoniae*. J Bacteriol. 2009;191(18):5859-64.
6. Slager J, Aprianto R, Veening JW. Deep genome annotation of the opportunistic human pathogen *Streptococcus pneumoniae* D39. Nucleic Acids Res. 2018;46(19):9971-89.
7. Edwards U, Rogall T, Blocker H, Emde M, Bottger EC. Isolation and direct complete nucleotide determination of entire genes. Characterization of a gene coding for 16S ribosomal RNA. Nucleic Acids Res. 1989;17(19):7843-53.
8. Weisburg WG, Barns SM, Pelletier DA, Lane DJ. 16S ribosomal DNA amplification for phylogenetic study. J Bacteriol. 1991;173(2):697-703.
9. Johnsborg O, Eldholm V, Bjørnstad ML, Håvarstein LS. A predatory mechanism dramatically increases the efficiency of lateral gene transfer in *Streptococcus pneumoniae* and related commensal species. Mol Microbiol. 2008;69(1):245-53.
